# A fractional version of Rivière’s GL(n)-gauge

**DOI:** 10.1007/s10231-021-01180-9

**Published:** 2021-12-14

**Authors:** Francesca Da Lio, Katarzyna Mazowiecka, Armin Schikorra

**Affiliations:** 1 Department of Mathematics, ETH Zürich, Rämistrasse 101, 8092 Zürich, Switzerland; 2 Lehrstuhl für Angewandte Analysis, RWTH Aachen University, Pontdriesch 14-16, 52062 Aachen, Germany; 3 Department of Mathematics, University of Pittsburgh, 301 Thackeray Hall, Pittsburgh, PA 15260 USA

**Keywords:** Fractional divergence, Fractional div-curl lemma, Fractional harmonic maps, 42B37, 42B30, 35R11, 58E20, 35B65

## Abstract

We prove that for antisymmetric vector field $\Omega$ with small $L^2$-norm there exists a gauge $A \in L^\infty \cap {\dot{W}}^{1/2,2}({\mathbb {R}}^1,GL(N))$ such that

$$\begin{aligned} {\text {div}}_{\frac{1}{2}} (A\Omega - d_{\frac{1}{2}} A) = 0. \end{aligned}$$

This extends a celebrated theorem by Rivière to the nonlocal case and provides conservation laws for a class of nonlocal equations with antisymmetric potentials, as well as stability under weak convergence.

## Introduction

In the celebrated work [27] Rivière showed that for two-dimensional disks $D \subset {\mathbb {R}}^2$ for any $\Omega \in L^2(D,so(N)\otimes \bigwedge \nolimits ^1 {\mathbb {R}}^2)$, i.e., $\Omega _{ij}=-\Omega _{ji} \in L^2(D,\bigwedge \nolimits ^1 {\mathbb {R}}^2)$ there exists a *GL*(*N*)-gauge, namely a matrix-valued function $A,A^{-1} \in L^\infty \cap W^{1,2}(D,GL(N))$ such that

$$\begin{aligned} {\text {div}}(A\Omega - \nabla A) = 0. \end{aligned}$$

These are distortions of the orthonormal Uhlenbeck’s Coulomb gauges, [36], namely $P \in L^\infty \cap W^{1,2}(D,SO(N))$ which satisfy

$$\begin{aligned} {\text {div}}(P\Omega P^t - P{^t}\nabla P) = 0. \end{aligned}$$

As Rivière showed in [27], the *GL*(*N*)-gauges have the advantage that they can transform equations of the form

1.1 $$\begin{aligned} -\Delta u = \Omega \cdot \nabla u \end{aligned}$$

into a conservation law

$$\begin{aligned} {\text {div}}(A \nabla u)={\text {div}}((\nabla A - A\Omega )u). \end{aligned}$$

This is important since (1.1) is the structure of the equation for harmonic maps, *H*-surfaces, and more generally the Euler–Lagrange equations of a large class of conformally invariant variational functionals. The *GL*(*N*)-gauge transform allows for regularity theory and the study of weak convergence [27]; it also is an important tool for energy quantization, see [16].

In recent years a theory of fractional harmonic maps has developed, beginning with the work by Rivière and the first named author, [9, 10]. bubbling analysis was initiated in [6]. Fractional harmonic maps have a variety of applications: they appear as free boundary of minimal surfaces or harmonic maps [8, 21, 24, 31]; they are also related to nonlocal minimal surfaces [22] and to knot energies [2, 3].

We recall that in [10] the first named author and Rivière considered nonlocal Schödinger-type systems of the form

1.2 $$\begin{aligned} (-\Delta )^{\frac{1}{4}} v=\Omega v\quad \quad \text{ in } {\mathcal {D}}'({\mathbb {R}}), \ \end{aligned}$$

where $\Omega$ is an antisymmetric potential in $L^2({\mathbb {R}},so(N))$, $v\in L^2({\mathbb {R}},{{\mathbb {R}}}^N)$. The main technique to establish the sub-criticality of systems (1.2) is to perform a *change of gauge* by rewriting them after having multiplied *v* by a well-chosen rotation-valued map $P\in {\dot{W}}^{1/2,2}({{\mathbb {R}}},SO(N))$ which is ”integrating” $\Omega$ in an optimal way. The key point in [9, 10] was the discovery of particular algebraic structures (three-term commutators) that play the role of the Jacobians in the case of local systems in 2-D with an antisymmetric potential and that enjoy suitable integrability by compensations properties. In [17] the second and the third named authors introduced a new approach to fractional harmonic maps by considering nonlocal systems with an antisymmetric potential which is seen itself as a nonlocal operator. As we will explain later, such an approach is similar in the spirit to that introduced by Hélein in [15] in the context of harmonic maps.

It begins with the definition of “nonlocal one forms”. $F \in L^p(\bigwedge \nolimits _{od}^1 {\mathbb {R}}^n)$ if $F: {\mathbb {R}}^n \times {\mathbb {R}}^n \rightarrow {\mathbb {R}}$ and

$$\begin{aligned} \int _{{\mathbb {R}}^n}\int _{{\mathbb {R}}^n} |F(x,y)|^p\, \frac{\,\mathrm {d}x\,\mathrm {d}y}{|x-y|^n} <\infty . \end{aligned}$$

The *s*-differential, which takes function $u: {\mathbb {R}}^n \rightarrow {\mathbb {R}}$ into 1-forms, is then given by

$$\begin{aligned} d_s u(x,y) :=\frac{u(x)-u(y)}{|x-y|^s}. \end{aligned}$$

The scalar product for two 1-forms, $F \in L^p(\bigwedge \nolimits _{od}^1 {\mathbb {R}}^n)$ and $G \in L^{p'}(\bigwedge \nolimits _{od}^1 {\mathbb {R}}^n)$, is then given by

$$\begin{aligned} F\cdot G(x) = \int _{{\mathbb {R}}^n} F(x,y) G(x,y) \frac{dy}{|x-y|^n}. \end{aligned}$$

The fractional divergence ${\text {div}}_s$, which takes 1-forms into functions, is then the formal adjoint to $d_s$, namely

$$\begin{aligned} {\text {div}}_s F[\varphi ] :=\int _{{\mathbb {R}}^n} F \cdot d_s \varphi \quad \forall \varphi \in C_c^\infty ({\mathbb {R}}^n). \end{aligned}$$

For more details we refer to Sect. 2. With this notation in mind we now consider equations of the form

1.3 $$\begin{aligned} {\text {div}}_{\frac{1}{2}} (d_{\frac{1}{2}} u) = \Omega \cdot d_{\frac{1}{2}} u\quad \text {in }{\mathbb {R}}, \end{aligned}$$

or in index form

$$\begin{aligned} {\text {div}}_{\frac{1}{2}} (d_{\frac{1}{2}} u^i) = \sum _{j=1}^N \Omega _{ij} \cdot d_{\frac{1}{2}} u^j \quad \text {in }{\mathbb {R}},\quad i=1,\ldots ,N, \end{aligned}$$

where $$u \in (L^2 + L^\infty )\cap \dot{W}^{\frac{1}{2},2}({\mathbb {R}},{\mathbb {R}}^N)$$ and $$\Omega _{ij} = - \Omega _{ji} \in L^2(\bigwedge \nolimits ^1_{od} {\mathbb {R}})$$.

The main observation in [17] is that the above notation and the above equation are not merely some random definitions of only analytical interest. Rather it was shown that the role of (1.3) for fractional harmonic maps is similar to the role of (1.1) for harmonic maps. In [17] it was shown that there exists a $div-curl$ lemma in the spirit of [5], that fractional harmonic maps into spheres satisfy a conservation law in the spirit of [15], and that fractional harmonic maps into spheres essentially satisfy equations of the form (1.3), in the spirit of [27], and that an analogue of Uhlenbeck’s gauge exist. In [20] this argument was further pushed to equations of stationary harmonic map in higher dimensional domains.

We mention that in [7] the authors found quasi conservation laws for nonlocal Schrödinger-type systems of the form

1.4 $$\begin{aligned} (-\Delta )^{1/4} v=\Omega v+g(x) \end{aligned}$$

where $v\in L^2({\mathbb {R}})$, $\Omega \in L^2({\mathbb {R}},so(N))$, and *g* is a tempered distribution. As we have already pointed out above, systems (1.4) represent a particular case of systems (1.3) studied in the present paper in the sense that the antisymmetric potential $\Omega$ in (1.4) is a pointwise function. The conservation laws found in [7] are a consequence of a stability property of some three-term commutators by the multiplication of $P \in SO(N)$ and also of the regularity results obtained previously for such commutators. The reformulation of (1.4) in terms of conservation laws has permitted to get the quantization in the neck regions of the $L^2$ norms of the negative part of sequences of solutions to systems of the type (1.4).

The conservation laws that we obtain in the current paper are more similar in the spirit to those found in the paper [27] for harmonic maps and concern nonlocal systems (1.3) where the antisymmetric potential acts in general as a nonlocal operator. We hope this technique to be as useful for the question of concentration compactness and energy quantization for systems as it was in the local case in [16]; a question we will study in a future work.

Applying a gauge $A \in L^\infty \cap {\dot{W}}^{\frac{1}{2},2}$ to the Eq. (1.3), we find (see Lemma 4.1),

$$\begin{aligned} {\text {div}}_{\frac{1}{2}} (A_{ik} d_{\frac{1}{2}} u^k) =\left( A_{i\ell }\Omega _{\ell k}\, -d_{\frac{1}{2}} A_{ik} \right) \cdot d_{\frac{1}{2}}u^k. \end{aligned}$$

Our main result is then the existence of the nonlocal analogue of Rivière’s *GL*(*N*)-Coulomb gauge [27], namely we have

### Theorem 1.1

There exists a number $0<\sigma \ll 1$ such that the following holds.

If $\Omega \in L^2(\bigwedge \nolimits _{od}^1{\mathbb {R}})$ is antisymmetric, i.e., $\Omega _{ij} = -\Omega _{ji}$ and satisfies

$$\begin{aligned} \Vert \Omega \Vert _{L^2(\bigwedge \nolimits _{od}^1 {\mathbb {R}})} < \sigma , \end{aligned}$$

then there exists an invertible matrix-valued function $A \in L^\infty \cap {\dot{W}}^{\frac{1}{2},2}({\mathbb {R}},GL(N))$ such that for $\Omega ^A :=A\Omega - d_\frac{1}{2} A$ we have

$$\begin{aligned} {\text {div}}_\frac{1}{2} \left( \Omega ^A \right) =0. \end{aligned}$$

Moreover, we have

1.5 $$\begin{aligned}{}[A]_{W^{\frac{1}{2},2}({\mathbb {R}})} \precsim \Vert \Omega \Vert _{L^2(\bigwedge \nolimits _{od}^1 {\mathbb {R}})}, \quad \Vert A\Vert _{L^\infty ({\mathbb {R}})}\precsim 1+ \Vert \Omega \Vert _{L^2(\bigwedge \nolimits _{od}^1 {\mathbb {R}})}. \end{aligned}$$

As an immediate corollary, we obtain

### Corollary 1.2

(Conservation law) Assume $u\in {\dot{W}}^{\frac{1}{2},2}({\mathbb {R}},{\mathbb {R}}^N)\cap (L^2+L^\infty )({\mathbb {R}},{\mathbb {R}}^N)$ and $f\in {\dot{W}}^{-\frac{1}{2},2}({\mathbb {R}},{\mathbb {R}}^N)$ satisfy

$$\begin{aligned} {\text {div}}_{\frac{1}{2}} (d_{\frac{1}{2}} {u}) = \Omega \cdot d_{\frac{1}{2}} u+f, ~~\text{ in } {\mathcal {D}}'({\mathbb {R}}) \end{aligned}$$

and $\Omega$ satisfies the condition of Theorem 1.1. Then there exists a matrix *A* such that for $\Omega ^A :=A\Omega - d_\frac{1}{2} A$ we have

$$\begin{aligned} {\text {div}}_{\frac{1}{2}} \left( A d_{\frac{1}{2}} u- {(\Omega ^A)^*} u \right) =Af, \quad ~~\text { in } {\mathcal {D}}'({\mathbb {R}}), \end{aligned}$$

where $${(\Omega ^A)^*(x,y):=\Omega ^A(y,x)}$$.

Theorem 1.1 is applicable to the half-harmonic map system as derived [17, Proposition 4.2], because of a localization result, see Proposition B.1.

With the methods of Theorem 1.1, we obtain the analogue of [27, Theorem I.5], our second main result.

### Theorem 1.3

Assume $\Omega _\ell \in L^2(\bigwedge \nolimits _{od}^1 {\mathbb {R}})$ is a sequence of antisymmetric vector fields, i.e., $(\Omega _{ij})_\ell = - (\Omega _{ji})_\ell$, weakly convergent in $L^2$ to an $\Omega \in L^2(\bigwedge \nolimits _{od}^1 {\mathbb {R}})$. Assume further that $f_\ell \in {\dot{W}}^{-\frac{1}{2},2}({\mathbb {R}}, {\mathbb {R}}^N)$ converges strongly to *f* in ${\dot{W}}^{-\frac{1}{2},2}$, and assume that $u_\ell \in (L^2+L^\infty ({\mathbb {R}}))\cap {\dot{W}}^{\frac{1}{2},2}({\mathbb {R}},{\mathbb {R}}^N)$ is a sequence of solutions to

1.6 $$\begin{aligned} (-\Delta )^{\frac{1}{2}} u_\ell = \Omega _{\ell } \cdot d_\frac{1}{2} u_\ell + f_\ell \quad ~~\text{ in }~ {\mathcal {D}}'({\mathbb {R}}) \end{aligned}$$

such that $\sup _{\ell }\left( \Vert u_\ell \Vert _{L^2+L^\infty ({\mathbb {R}})}+[u_\ell ]_{W^{\frac{1}{2},2}({\mathbb {R}})} \right) <\infty$. Then, up to taking a subsequence $u_\ell$ converges weakly in ${\dot{W}}^{\frac{1}{2},2}({\mathbb {R}},{\mathbb {R}}^N)$ to some $u\in {\dot{W}}^{\frac{1}{2},2}({\mathbb {R}},{\mathbb {R}}^N) \cap ((L^2 + L^\infty )({\mathbb {R}},{\mathbb {R}}^N))$, which is a solution to

$$\begin{aligned} (-\Delta )^\frac{1}{2} u = \Omega \cdot d_\frac{1}{2} u + f ~~\text{ in } ~{\mathcal {D}}'({\mathbb {R}}). \end{aligned}$$

Here, as usual, we denote

$$\begin{aligned} \Vert f\Vert _{L^2+L^\infty ({\mathbb {R}})} = \inf _{f_1 \in L^2({\mathbb {R}})} \left( \Vert f_1\Vert _{L^2({\mathbb {R}})} + \Vert f-f_1\Vert _{L^\infty ({\mathbb {R}})} \right) . \end{aligned}$$

Theorem 1.3 will be proven in Sect. 4.

## Preliminaries and useful tools

We follow the notation of [17] for the nonlocal operators. For readers convenience we recall it here. We write ${\mathcal {M}}({\mathbb {R}}^n)$ for the space of all functions $f:{\mathbb {R}}^n \rightarrow {\mathbb {R}}$ measurable with respect to the Lebesgue measure $\,\mathrm {d}x$ and ${\mathcal {M}}(\bigwedge \nolimits _{od}^1{\mathbb {R}}^n)$ for the space of vector fields $F:{\mathbb {R}}^n \times {\mathbb {R}}^n \rightarrow {\mathbb {R}}$ measurable with respect to the $\frac{\,\mathrm {d}x\,\mathrm {d}y}{|x-y|^n}$ measure, where “*od*" stands for “off diagonal”.

For two vector fields $F,\, G \in {\mathcal {M}}(\bigwedge \nolimits _{od}^1 {\mathbb {R}}^n)$, the scalar product is defined as

$$\begin{aligned} F \cdot G (x) :=\int _{{\mathbb {R}}^n} F(x,y) \, G(x,y) \frac{\,\mathrm {d}y}{|x-y|^n}. \end{aligned}$$

For any $p>1$ the natural $L^p$-space on vector fields $F:{\mathbb {R}}^n\times {\mathbb {R}}^n \rightarrow {\mathbb {R}}$ is induced by the norm

$$\begin{aligned} \Vert F\Vert _{L^p(\bigwedge \nolimits _{od}^1 {\mathbb {R}}^n)} :=\left( \int _{{\mathbb {R}}^n} \int _{{\mathbb {R}}^n} |F(x,y)|^p\frac{\,\mathrm {d}x \,\mathrm {d}y}{|x-y|^n} \right) ^\frac{1}{p} \end{aligned}$$

and for $D\subset {\mathbb {R}}^n$ we define

$$\begin{aligned} \Vert F\Vert _{L^p(\bigwedge \nolimits _{od}^1 D)} :=\left( \iint _{(D\times {\mathbb {R}}^n)\cup ({\mathbb {R}}^n\times D)} |F(x,y)|^p \frac{\,\mathrm {d}x \,\mathrm {d}y}{|x-y|^n} \right) ^\frac{1}{p}. \end{aligned}$$

Let $s\in (0,1)$. For $f:{\mathbb {R}}^n \rightarrow {\mathbb {R}}$ we let the *s*-gradient $d_s :{\mathcal {M}}({\mathbb {R}}^n)\rightarrow {\mathcal {M}}(\bigwedge \nolimits _{od}^1 {\mathbb {R}}^n)$ to be

$$\begin{aligned} d_s f(x,y) :=\frac{f(x) - f(y)}{|x-y|^s}. \end{aligned}$$

Observe that with this notation we have

$$\begin{aligned} \Vert d_s f\Vert _{L^p(\bigwedge \nolimits _{od}^1 {\mathbb {R}}^n)} = [f]_{W^{s,p}({\mathbb {R}}^n)}, \end{aligned}$$

where

$$\begin{aligned} {[}f]_{W^{s,p}({\mathbb {R}}^n)}=\left( \int _{{\mathbb {R}}^n}\int _{{\mathbb {R}}^n}\frac{|f(x)-f(y)|^p}{|x-y|^{n+sp}}\,\mathrm {d}x\,\mathrm {d}y\right) ^{1/p} \end{aligned}$$

is the Gagliardo–Slobodeckij seminorm.

Let $s\in (0.1)$ and $F\in {\mathcal {M}}(\bigwedge \nolimits _{od}^1 {\mathbb {R}}^n)$. We define the fractional *s*-divergence in the distributional way

$$\begin{aligned} {\text {div}}_{s} F[\varphi ] :=\int _{{\mathbb {R}}^n} \int _{{\mathbb {R}}^n} F(x,y)\, d_s \varphi (x,y) \frac{\,\mathrm {d}x\,\mathrm {d}y}{|x-y|^n}, \quad \varphi \in C_c^\infty ({\mathbb {R}}^n), \end{aligned}$$

whenever the integrals converge.

With this notation we have ${\text {div}}_s d_s = (-\Delta )^s$, i.e.,

$$\begin{aligned} \int _{{\mathbb {R}}^n} d_s f \cdot d_s g(x) \,\mathrm {d}x= {\frac{2}{C_{n,s}}} \int _{{\mathbb {R}}} (-\Delta )^s f(x) g(x) \,\mathrm {d}x, \end{aligned}$$

where the fractional Laplacian is defined as

$$\begin{aligned} (-\Delta )^s f (x) :={C_{n,s}} P.V. \int _{{\mathbb {R}}^n} \frac{f(x)- f(y)}{|x-y|^{2s}}\frac{\,\mathrm {d}y}{|x-y|^n}. \end{aligned}$$

A simple observation is the following

### Lemma 2.1

Let $F \in {\mathcal {M}}(\bigwedge \nolimits ^1_{od} {\mathbb {R}}^n)$ then we define

$$\begin{aligned} F^*(x,y) :={F(y,x)}. \end{aligned}$$

If $${\text {div}}_s F = 0$$ then $${\text {div}}_s F^*= 0$$.

Moreover, for any $F \in {\mathcal {M}}(\bigwedge \nolimits ^1_{od} {\mathbb {R}}^n)$ and $u\in {\mathcal {M}}({\mathbb {R}}^n)$ we have

2.1 $$\begin{aligned} {\text {div}}_{s} (Fu(x)) = {\text {div}}_s(F) u+F^*\cdot d_{s}u \end{aligned}$$

and

2.2 $$\begin{aligned} {\text {div}}_{s} (Fu(y)) = {\text {div}}_s(F) u-F \cdot d_{s}u \end{aligned}$$

whenever each term is well-defined.

### Proof

We have

$$\begin{aligned} F(x,y) u(x) (\varphi (x)-\varphi (y)) = F(x,y) (u(x)\varphi (x)-u(y)\varphi (y)) - F(x,y) (u(x)-u(y))\varphi (y). \end{aligned}$$

Thus,

2.3 $$\begin{aligned} \begin{aligned} {{\text {div}}_s( Fu(x))[\varphi ] }&=\int _{{\mathbb {R}}^n} \int _{{\mathbb {R}}^n} \frac{F(x,y) u(x) (\varphi (x)-\varphi (y))}{|x-y|^{n+s}}\, \,\mathrm {d}y\,\mathrm {d}x\\&= \int _{{\mathbb {R}}^n} \int _{{\mathbb {R}}^n} \frac{F(x,y) (u(x)\varphi (x)-u(y)\varphi (y))}{|x-y|^{n+s}}\, \,\mathrm {d}y\,\mathrm {d}x\\&\quad - \int _{{\mathbb {R}}^n} \int _{{\mathbb {R}}^n} \frac{F(x,y) (u(x)-u(y))\varphi (y) }{|x-y|^{n+s}}\, \,\mathrm {d}y\,\mathrm {d}x. \end{aligned} \end{aligned}$$

As for the latter term, we have

2.4 $$\begin{aligned} \begin{aligned} -&\int _{{\mathbb {R}}^n} \int _{{\mathbb {R}}^n} \frac{F(x,y) (u(x)-u(y))\varphi (y)}{|x-y|^{n+s}} \,\mathrm {d}y\,\mathrm {d}x\\&={-}\int _{{\mathbb {R}}^n} \int _{{\mathbb {R}}^n} \frac{-F(y,x) (u(x)-u(y))\varphi (x)}{|x-y|^{n+s}} \,\mathrm {d}y\,\mathrm {d}x\\&= \int _{{\mathbb {R}}^n} \int _{{\mathbb {R}}^n} \frac{F^*(x,y) (u(x)-u(y))\varphi (x)}{|x-y|^{n+s}} \,\mathrm {d}y\,\mathrm {d}x. \end{aligned} \end{aligned}$$

Combining (2.3) with (2.4), we obtain (2.1). The proof of (2.2) is similar. $\square$

We also denote

$$\begin{aligned} |D_{s,q} f|(x) :=\left( \int _{{\mathbb {R}}^n}\frac{|f(x) - f(y)|^q}{|x-y|^{n+sq}} \,\mathrm {d}y \right) ^\frac{1}{q}. \end{aligned}$$

We will be using the following “Sobolev embedding” theorem.

### Theorem 2.2

Let $s\in (0,1)$, $t\in (s,1)$, and let $p,\,p^*>1$ satisfy

$$\begin{aligned} s-\frac{n}{p^*} = t - \frac{n}{p}, \end{aligned}$$

where $q>1$ with $p^*>\frac{nq}{n+sq}$. Then we have

2.5 $$\begin{aligned} \Vert |{\mathcal {D}}_{s,q}f|\Vert _{L^{p^*}({\mathbb {R}}^n)} \precsim \Vert (-\Delta )^{\frac{t}{2}} f\Vert _{L^p({\mathbb {R}}^n)} \end{aligned}$$

and for any $r\in [1,\infty ]$

2.6 $$\begin{aligned} \Vert |{\mathcal {D}}_{s,q}f|\Vert _{L^{(p^*,r)}({\mathbb {R}}^n)} \precsim \Vert (-\Delta )^{\frac{t}{2}} f\Vert _{L^{(p,r)}({\mathbb {R}}^n)}. \end{aligned}$$

For the proof see Appendix C.

We will also need the following Wente’s inequality from [17].

### Lemma 2.3

([17, Corollary 2.3]) Let $s\in (0,1)$, $p>1$, and let $p'$ be the Hölder conjugate of *p*. Assume moreover that $F'\in L^p(\bigwedge \nolimits _{od}^1 {\mathbb {R}})$ and $g\in W^{s,p'}({\mathbb {R}})$ with ${\text {div}}_s F=0$. Let *R* be a linear operator such that for some $\Lambda >0$ satisfies

$$\begin{aligned} |R[\varphi ]| \le \Lambda \Vert (-\Delta )^{\frac{1}{4}} \varphi \Vert _{L^{(2,\infty )}({\mathbb {R}})}, \end{aligned}$$

where $L^{(2,\infty )}({\mathbb {R}})$ denote the weak $L^2$ space. Then any distributional solution $u\in {\dot{W}}^{\frac{1}{2},2}({\mathbb {R}})$ to

$$\begin{aligned} (-\Delta )^{\frac{1}{2}}u = F \cdot d_s g + R \quad \text {in } {\mathbb {R}}\end{aligned}$$

is continuous. Moreover, if $\lim _{x \rightarrow \pm \infty } |u(x)|=0$, then we have the estimate

2.7 $$\begin{aligned} \Vert u\Vert _{L^\infty ({\mathbb {R}})}+\Vert d_\frac{1}{2} u\Vert _{L^2(\bigwedge \nolimits _{od}^1{\mathbb {R}})} \precsim \Vert F\Vert _{L^p(\bigwedge \nolimits _{od}^1 {\mathbb {R}})} \Vert d_s g\Vert _{L^{p'}(\bigwedge \nolimits _{od}^1 {\mathbb {R}})} + \Lambda . \end{aligned}$$

Our proof will also be based on the following choice of a good gauge.

### Theorem 2.4

([17, Theorem 4.4]) For $\Omega _{ij} = -\Omega _{ji} \in L^2(\bigwedge \nolimits ^1_{od}{\mathbb {R}})$, there exists $P \in {\dot{W}}^{\frac{1}{2}}({\mathbb {R}},SO(N))$ such that

$$\begin{aligned} {\text {div}}_{\frac{1}{2}} \Omega ^P_{ij} = 0 \quad \text{ for } \text{ all } i,j \in \{1,\ldots ,N\}, \end{aligned}$$

where

$$\begin{aligned} \Omega ^P = \frac{1}{2} \left( d_{\frac{1}{2}}P(x,y) \left( P^T(y) + P^T(x) \right) - P(x) \Omega (x,y) P^T(y) - P(y) \Omega (x,y) P^T(x) \right) \end{aligned}$$

and

2.8 $$\begin{aligned}{}[P]_{W^{\frac{1}{2},2}({\mathbb {R}})} \precsim \Vert \Omega \Vert _{L^2(\bigwedge \nolimits _{od}^1 {\mathbb {R}})}. \end{aligned}$$

## Proof of Theorem 1.1

In this section we prove Theorem 1.1. We will be looking for an *A* in the form $A=(I+\varepsilon )P$, where *P* is chosen to be the good gauge from Theorem 2.4. The idea to take perturbation of rotations of the form $(I+\varepsilon )P$ has been taken from [28] in the context of local Schrödinger equations with antisymmetric potentials. This has been also exploited in [7].

### Lemma 3.1

Assume that $$A = (I+\varepsilon ) P$$.

Then for

$$\begin{aligned} \Omega ^P(x,y) = \frac{1}{2} \left( d_{\frac{1}{2}}P(x,y) \left( P^T(y) + P^T(x) \right) - P(x) \Omega (x,y) P^T(y) - P(y) \Omega (x,y) P^T(x) \right) \end{aligned}$$

we have

$$\begin{aligned} A(x)\Omega (x,y)\, -d_{\frac{1}{2}} A(x,y) =- (I+\varepsilon (x))\, \Omega ^P(x,y)\, P(y) - d_{\frac{1}{2}} \varepsilon (x,y)\, P(y) +R_\varepsilon (x,y), \end{aligned}$$

where $R_\varepsilon$ is given by the formula

3.1 $$\begin{aligned} \begin{aligned} R_\varepsilon (x,y) :=&\frac{1}{2} (I+\varepsilon (x)) \bigg ( d_{\frac{1}{4}} P(x,y)\, d_{\frac{1}{4}} P^T(x,y) \\&-P(x)\, \Omega (x,y)\, \left( P^T(x)-P^T(y) \right) \\&+\left( P(x)-P(y) \right) \,\Omega (x,y)\, P^T(x) \bigg ) P(y). \end{aligned} \end{aligned}$$

### Proof

Recall that

$$\begin{aligned} d_\frac{1}{2}(fg)(x,y) = d_\frac{1}{2} f(x,y)\, g(y) + f(x) d_\frac{1}{2} g(x,y). \end{aligned}$$

Thus, applying this to $d_\frac{1}{2} ((I+\varepsilon )P)(x,y)$ we get

3.2 $$\begin{aligned} \begin{aligned}&A(x)\Omega (x,y) - d_\frac{1}{2} A(x,y)\\&\quad =(I+\varepsilon (x)) P(x)\Omega (x,y)\, -d_{\frac{1}{2}} \left( (I+\varepsilon ) P \right) (x,y)\\&\quad =(I+\varepsilon (x)) \left( P(x)\, \Omega (x,y)\, -d_{\frac{1}{2}} P(x,y) \right) -d_{\frac{1}{2}} \varepsilon (x,y)\, P(y) \\&\quad =-(I+\varepsilon (x)) \left( d_{\frac{1}{2}} P(x,y)\, P^T(y)-P(x)\, \Omega (x,y)\, P^T(y) \right) P(y) -d_{\frac{1}{2}} \varepsilon (x,y)\, P(y). \end{aligned} \end{aligned}$$

Next we observe that

3.3 $$\begin{aligned} \begin{aligned}&d_{\frac{1}{2}} P(x,y)\, P^T(y)-P(x)\, \Omega (x,y)\, P^T(y)\\&\quad =\frac{1}{2}\left( d_{\frac{1}{2}} P(x,y)\, \left( P^T(x)+P^T(y) \right) -P(x)\, \Omega (x,y)\, P^T(y)-P(y)\,\Omega (x,y)\, P^T(x) \right) \\&\qquad -\frac{1}{2} \Big (d_{\frac{1}{2}} P(x,y)\, \left( P^T(x)-P^T(y) \right) -P(x)\, \Omega (x,y)\, \left( P^T(x)-P^T(y) \right) \\&\qquad +\left( P(x)-P(y) \right) \,\Omega (x,y)\, P^T(x)\Big ). \end{aligned} \end{aligned}$$

That is, plugging in (3.3) into (3.2) we get the claim for

$$\begin{aligned} \begin{aligned} R_\varepsilon (x,y) :&=\frac{1}{2} (I+\varepsilon (x)) \bigg ( d_{\frac{1}{4}} P(x,y)\, d_{\frac{1}{4}} P^T(x,y) \\&-P(x)\, \Omega (x,y)\, \left( P^T(x)-P^T(y) \right) \\&+\left( P(x)-P(y) \right) \,\Omega (x,y)\, P^T(x) \bigg ) P(y). \end{aligned} \end{aligned}$$

$$\square $$

### Lemma 3.2

Assume that we have $\varepsilon \in L^\infty \cap {\dot{W}}^{1/2,2}({\mathbb {R}}),\, a\in {\dot{W}}^{1/2,2}({\mathbb {R}})$, and $B\in L^2(\bigwedge \nolimits _{od}^1 {\mathbb {R}})$ satisfying the equations

3.4 $$\begin{aligned} - (I+\varepsilon (x))\, \Omega ^P(x,y)\, P(y) - d_{\frac{1}{2}} \varepsilon (x,y)\, P(y) +R_\varepsilon (x,y) = d_\frac{1}{2} a(x,y) + B(x,y) \end{aligned}$$

and

3.5 $$\begin{aligned} \begin{aligned}&- {\text {div}}_{\frac{1}{2}}\left( (I+\varepsilon (x))\, \Omega ^P(x,y) \right) - {\text {div}}_{\frac{1}{2}}\left( d_{\frac{1}{2}} \varepsilon (x,y) \right) +{\text {div}}_{\frac{1}{2}}(R_\varepsilon (x,y) P^T(y))\\&\quad = {\text {div}}_{\frac{1}{2}} \left( B(x,y) P^T(y) \right) , \end{aligned} \end{aligned}$$

with

3.6 $$\begin{aligned}{}[P]_{W^{1/2,2}({\mathbb {R}})} < \sigma . \end{aligned}$$

Then, for sufficiently small $\sigma$ we have $a=const$.

### Proof

We multiply (3.4) by $P^T(y)$ from the right and take the $\frac{1}{2}$-divergence on both sides; then subtracting (3.5), we obtain

3.7 $$\begin{aligned} {\text {div}}_\frac{1}{2} (d_\frac{1}{2} a(x,y)P^T(y)) = 0. \end{aligned}$$

We use nonlocal Hodge decomposition Lemma A.1 and get the existence of functions ${\tilde{a}} \in {\dot{W}}^{\frac{1}{2},2}({\mathbb {R}})$, ${\tilde{B}}\in L^2(\bigwedge \nolimits _{od}^1 {\mathbb {R}})$ such that

3.8 $$\begin{aligned} d_\frac{1}{2} a(x,y)P^T(y) = d_\frac{1}{2} {\tilde{a}} (x,y) + {\tilde{B}}(x,y), \end{aligned}$$

and (recall $$|P|=1$$)

3.9 $$\begin{aligned} {\text {div}}_\frac{1}{2} {\tilde{B}} =0 \quad \text {and } \quad \Vert {\tilde{B}}\Vert _{L^2(\bigwedge \nolimits _{od}^1 {\mathbb {R}})} \precsim \Vert d_\frac{1}{2} a\Vert _{L^2(\bigwedge \nolimits _{od}^1 {\mathbb {R}})}. \end{aligned}$$

Thus, taking the $\frac{1}{2}$-divergence in (3.8) we obtain

$$\begin{aligned} 0 = {\text {div}}_\frac{1}{2} (d_\frac{1}{2} a(x,y)P^T(y)) = {\text {div}}_\frac{1}{2} (d_\frac{1}{2} {\tilde{a}} (x,y) + {\tilde{B}}(x,y)) = {\text {div}}_\frac{1}{2} (d_\frac{1}{2} {\tilde{a}}) = (-\Delta )^{\frac{1}{2}} {\tilde{a}}. \end{aligned}$$

This gives $(-\Delta )^{\frac{1}{2}}{\tilde{a}} =0$, thus ${\tilde{a}}$ is constant and without loss of generality we can take ${\tilde{a}} = 0$, see also [11, Theorem 1.1]. Thus, (3.8) becomes

$$\begin{aligned} d_\frac{1}{2} a(x,y)P^T(y) = {\tilde{B}}(x,y). \end{aligned}$$

That is

$$\begin{aligned} d_\frac{1}{2} a(x,y) = {\tilde{B}}(x,y) P(y). \end{aligned}$$

Taking the $\frac{1}{2}$-divergence, we obtain by Lemma 2.1

3.10 $$\begin{aligned} (-\Delta )^{\frac{1}{2}} a = {-{\tilde{B}}} \cdot d_\frac{1}{2} P, \end{aligned}$$

since on the right-hand side we have a *div-curl* term we can apply fractional Wente’s inequality, Lemma 2.3, and obtain from (2.7)

$$\begin{aligned} \Vert d_\frac{1}{2} a\Vert _{L^2(\bigwedge \nolimits _{od}^1 {\mathbb {R}})} \precsim \Vert {\tilde{B}}\Vert _{L^2(\bigwedge \nolimits _{od}^1 {\mathbb {R}})} \Vert d_\frac{1}{2} P\Vert _{L^2(\bigwedge \nolimits _{od}^1 {\mathbb {R}})}. \end{aligned}$$

Combining this with (3.9) and (3.6), we get

$$\begin{aligned} \Vert d_\frac{1}{2} a\Vert _{L^2(\bigwedge \nolimits _{od}^1 {\mathbb {R}})} \precsim \sigma \Vert d_\frac{1}{2} a\Vert _{L^2(\bigwedge \nolimits _{od}^1 {\mathbb {R}})}, \end{aligned}$$

which implies for sufficiently small $\sigma$ that

$$\begin{aligned} \Vert d_\frac{1}{2} a\Vert _{L^2(\bigwedge \nolimits _{od}^1 {\mathbb {R}})} = [a]_{W^{1/2,2}({\mathbb {R}})}=0 \end{aligned}$$

and thus $$a \equiv const$$. $$\square $$

Now we will focus on showing that there exists a solution to the Eqs. (3.4) and (3.5). We will do this by using the Banach fixed point theorem.

### Proposition 3.3

Let $\Omega \in L^2(\bigwedge \nolimits _{od}^1 {\mathbb {R}})$ be antisymmetric. There is a number $0<\sigma \ll 1$ such that the following holds:

Take $P\in {\dot{W}}^{\frac{1}{2},2}({\mathbb {R}},SO(N))$ and $\Omega ^P\in L^2(\bigwedge \nolimits _{od}^1{\mathbb {R}})$ from Theorem 2.4. Let us assume that

3.11 $$\begin{aligned}{}[P]_{W^{1/2,2}({\mathbb {R}})} + \Vert \Omega \Vert _{L^2(\bigwedge \nolimits _{od}^1 {\mathbb {R}})} < \sigma . \end{aligned}$$

Then, there exist $\varepsilon \in L^\infty \cap {\dot{W}}^{1/2,2}({\mathbb {R}})$, $a\in {\dot{W}}^{1/2,2}({\mathbb {R}})$, and $B\in L^2(\bigwedge \nolimits _{od}^1 {\mathbb {R}})$ that solve the equations

3.12 $$\begin{aligned} \left\{ \begin{array}{l} - (I+\varepsilon (x))\, \Omega ^P(x,y) P(y) {-} d_{\frac{1}{2}} \varepsilon (x,y) P(y) +R_\varepsilon (x,y) = d_\frac{1}{2} a(x,y) + B(x,y) \\ - {\text {div}}_{\frac{1}{2}}\left( (I+\varepsilon (x)) \Omega ^P(x,y) \right) {-} {\text {div}}_{\frac{1}{2}}(d_{\frac{1}{2}} \varepsilon (x,y)) +{\text {div}}_{\frac{1}{2}}(R_\varepsilon (x,y) P^T(y)) = {\text {div}}_{\frac{1}{2}} \left( B P^T(y) \right) , \end{array} \right. \end{aligned}$$

where $R_\varepsilon$ is defined in (3.1).

Moreover, $\varepsilon$ satisfies the estimate

3.13 $$\begin{aligned} \Vert \varepsilon \Vert _{L^\infty ({\mathbb {R}})} + [\varepsilon ]_{W^{\frac{1}{2},2}({\mathbb {R}})} \precsim \Vert \Omega \Vert _{L^2(\bigwedge \nolimits _{od}^1 {\mathbb {R}})}. \end{aligned}$$

We will need the following remainder terms estimates.

### Lemma 3.4

We have the following estimates

3.14 $$\begin{aligned} \begin{aligned}&\left| {\text {div}}_{\frac{1}{2}} (R_\varepsilon P^T{(y)})[\varphi ] \right| \\&\quad \precsim (1+\Vert \varepsilon \Vert _{L^\infty ({\mathbb {R}})}) (\Vert \Omega \Vert _{L^2(\bigwedge \nolimits _{od}^1{\mathbb {R}})} + [P]_{W^{1/2,2}({\mathbb {R}})})[P]_{W^{1/2,2}({\mathbb {R}})}\, \Vert (-\Delta )^{\frac{1}{4}}\varphi \Vert _{L^{(2,\infty )}({\mathbb {R}})} \end{aligned} \end{aligned}$$

and

3.15 $$\begin{aligned} \begin{aligned}&\left| {\text {div}}_{\frac{1}{2}} \left( (R_{\varepsilon _1} - R_{\varepsilon _2}) P^T{(y)} \right) [\varphi ] \right| \\&\quad \precsim \Vert \varepsilon _1-\varepsilon _2\Vert _{L^\infty ({\mathbb {R}})} (\Vert \Omega \Vert _{L^2(\bigwedge \nolimits _{od}^1{\mathbb {R}})} + [P]_{W^{1/2,2}({\mathbb {R}})})[P]_{W^{1/2,2}({\mathbb {R}})}\, \Vert (-\Delta )^{\frac{1}{4}}\varphi \Vert _{L^{(2,\infty )}({\mathbb {R}})}. \end{aligned} \end{aligned}$$

### Proof

We observe that for any $\varphi \in C_c^\infty ({\mathbb {R}})$ we have

3.16 $$\begin{aligned} \begin{aligned}&\left| {\text {div}}_{\frac{1}{2}}(R_\varepsilon P^T{(y)})[\varphi ] \right| \\&\quad \precsim \left| \int _{\mathbb {R}}\int _{\mathbb {R}}(I+\varepsilon (x)) \left( d_{\frac{1}{4}} P(x,y)\, d_{\frac{1}{4}} P^T(x,y) \right) \ d_{\frac{1}{2}} \varphi (x,y) \frac{\,\mathrm {d}x\,\mathrm {d}y}{|x-y|} \right| \\&\qquad +\left| \int _{\mathbb {R}}\int _{\mathbb {R}}(I+\varepsilon (x)) \left( P(x)\, \Omega (x,y)\, \left( P^T(x)-P^T(y) \right) \ d_{\frac{1}{2}} \varphi (x,y)\frac{\,\mathrm {d}x\,\mathrm {d}y}{|x-y|} \right) \right| \\&\qquad +\left| \int _{\mathbb {R}}\int _{\mathbb {R}}(I+\varepsilon (x))\left( P(x)-P(y) \right) \,\Omega (x,y)\, P^T(x) \ d_{\frac{1}{2}} \varphi (x,y)\frac{\,\mathrm {d}x\,\mathrm {d}y}{|x-y|} \right| \\&\quad \precsim \left( 1+\Vert \varepsilon \Vert _{L^\infty } \right) \, \int _{\mathbb {R}}\int _{\mathbb {R}}\left( |d_{\frac{1}{4}} P(x,y)|^2\, |d_{\frac{1}{2}} \varphi (x,y)|\,\right. \\&\qquad \left. +|\Omega (x,y)|\, |d_{\frac{1}{4}}P(x,y)|\, | d_{\frac{1}{4}} \varphi (x,y)|\right) \frac{\,\mathrm {d}x\,\mathrm {d}y}{|x-y|}\\&\quad =\left( 1+\Vert \varepsilon \Vert _{L^\infty } \right) \, \left( \mathcal {I} + \mathcal {II} \right) . \end{aligned} \end{aligned}$$

Let ${\mathcal {M}}$ be the Hardy–Littlewood maximal function and let $\alpha \in (0,1)$. We will use the following fractional counterpart (for the proof see [31, Proposition 6.6])

3.17 $$\begin{aligned} |f(x) - f(y)| \precsim |x-y|^\alpha \left( {\mathcal {M}} ((-\Delta )^{\frac{\alpha }{2}}f)(x) + {\mathcal {M}} ((-\Delta )^{\frac{\alpha }{2}}f)(y) \right) \end{aligned}$$

of the well-known inequality, see [4, 14]

$$\begin{aligned} |f(x) - f(y)| \precsim |x-y| \left( {\mathcal {M}}|\nabla f|(x) + {\mathcal {M}} |\nabla f|(y) \right) . \end{aligned}$$

We begin with the estimate of the first term on the right-hand side of (3.16).

We observe that by (3.17) and by the symmetry of the integrals we obtain

3.18 $$\begin{aligned} \begin{aligned}&{\mathcal {I}}:=\int _{\mathbb {R}}\int _{\mathbb {R}}|d_\frac{1}{4} P(x,y)|^2 |d_\frac{1}{2} \varphi (x,y)|\frac{\,\mathrm {d}x\,\mathrm {d}y}{|x-y|} \\&\quad \precsim \int _{{\mathbb {R}}} |{\mathcal {M}} ((-\Delta )^{\frac{1}{4}}\varphi )(x)| \int _{\mathbb {R}}|d_\frac{1}{4} P(x,y)|^2 \frac{{\,\mathrm {d}y\,\mathrm {d}x}}{|x-y|}. \end{aligned} \end{aligned}$$

Applying Hölder’s inequality (for Lorentz spaces), we obtain

3.19 $$\begin{aligned} \begin{aligned} \int _{{\mathbb {R}}} |{\mathcal {M}} ((-\Delta )^{\frac{1}{4}}\varphi )(x)| \int _{\mathbb {R}}|d_\frac{1}{4} P(x,y)|^2 \frac{{\,\mathrm {d}y\,\mathrm {d}x}}{|x-y|}&\precsim \Vert (-\Delta )^{\frac{1}{4}}\varphi \Vert _{L^{(2,\infty )}} \Vert |{\mathcal {D}}_{\frac{1}{4},2} P|^2\Vert _{L^{(2,1)}}\\&= \Vert (-\Delta )^{\frac{1}{4}}\varphi \Vert _{L^{(2,\infty )}} \Vert |{\mathcal {D}}_{\frac{1}{4},2} P|\Vert ^2_{L^{(4,2)}}, \end{aligned} \end{aligned}$$

where we used the notation from Sect. 2: for $s\in (0,1)$ and $q>1$ we write

$$\begin{aligned} |{\mathcal {D}}_{s,q}f|(x) :=\left( \int _{\mathbb {R}}\frac{|f(x)-f(y)|^q}{|x-y|^{1+sq}} \,\mathrm {d}y \right) ^\frac{1}{q}. \end{aligned}$$

Applying Theorem 2.2, (2.6) for $t=\frac{1}{2}$, we get

3.20 $$\begin{aligned} \Vert |{\mathcal {D}}_{\frac{1}{4},2} P|\Vert ^2_{L^{(4,2)}} \precsim \Vert (-\Delta )^{\frac{1}{4}}P\Vert ^2_{L^{(2,2)}} \precsim \Vert (-\Delta )^{\frac{1}{4}}P\Vert ^2_{L^2} = [P]_{W^{1/2,2}}^2. \end{aligned}$$

Thus, combining (3.18), (3.19), and (3.20) we obtain

3.21 $$\begin{aligned} {\mathcal {I}} = \int _{\mathbb {R}}\int _{\mathbb {R}}|d_\frac{1}{3} P(x,y)|^2 |d_\frac{1}{3} \varphi (x,y)|\frac{\,\mathrm {d}x\,\mathrm {d}y}{|x-y|} \precsim [P]_{W^{1/2,2}({\mathbb {R}})}^2 \Vert (-\Delta )^{\frac{1}{4}}\varphi \Vert _{L^{(2,\infty )}({\mathbb {R}})}. \end{aligned}$$

As for the second term of (3.16), we have

3.22 $$\begin{aligned} \begin{aligned} \mathcal {II}&:=\int _{\mathbb {R}}\int _{\mathbb {R}}|\Omega (x,y)||d_\frac{1}{4} P(x,y)||d_\frac{1}{4} \varphi (x,y)|\frac{\,\mathrm {d}x\,\mathrm {d}y}{|x-y|}\\&\precsim \Vert \Omega \Vert _{L^2(\bigwedge \nolimits _{od}^1 {\mathbb {R}})} \left( \int _{{\mathbb {R}}} \int _{{\mathbb {R}}} |d_\frac{1}{4} P(x,y)|^2 |d_\frac{1}{4} \varphi (x,y)|^2 \frac{\,\mathrm {d}x \,\mathrm {d}y}{|x-y|} \right) ^\frac{1}{2}. \end{aligned} \end{aligned}$$

Applying once again (3.17), we obtain

3.23 $$\begin{aligned} \begin{aligned}&\int _{{\mathbb {R}}} \int _{{\mathbb {R}}} |d_\frac{1}{4} P(x,y)|^2 |d_\frac{1}{4} \varphi (x,y)|^2 \frac{\,\mathrm {d}x \,\mathrm {d}y}{|x-y|} \\&\quad \precsim \int _{\mathbb {R}}\int _{\mathbb {R}}\left( {\mathcal {M}} ((-\Delta )^{\frac{1}{8}}\varphi )(x) + {\mathcal {M}} ((-\Delta )^{\frac{1}{8}}\varphi ) (y) \right) ^2 |d_\frac{1}{4} P(x,y)|^2 \frac{\,\mathrm {d}x \,\mathrm {d}y}{|x-y|}\\&\quad \precsim \int _{\mathbb {R}}\left( {\mathcal {M}} ((-\Delta )^{\frac{1}{8}}\varphi )(x) \right) ^2 \int _{\mathbb {R}}|d_\frac{1}{4} P(x,y)|^2 \frac{{\,\mathrm {d}y \,\mathrm {d}x }}{|x-y|}. \end{aligned} \end{aligned}$$

Using Hölder’s inequality and then Sobolev embedding, we get

3.24 $$\begin{aligned} \begin{aligned}&\int _{\mathbb {R}}\left( {\mathcal {M}} ((-\Delta )^{\frac{1}{8}}\varphi )(x) \right) ^2 \int _{\mathbb {R}}|d_\frac{1}{4} P(x,y)|^2 \frac{{ \,\mathrm {d}y \,\mathrm {d}x}}{|x-y|}\\&\quad \precsim \Vert ({\mathcal {M}} (-\Delta )^\frac{1}{8} \varphi )^2 \Vert _{L^{(2,\infty )}({\mathbb {R}})} \Vert |{\mathcal {D}}_{\frac{1}{4},2} P|^2\Vert _{L^{(2,1)}({\mathbb {R}})}\\&\quad \precsim \Vert (-\Delta )^\frac{1}{8} \varphi \Vert ^2_{L^{(4,\infty )}({\mathbb {R}})} \Vert |{\mathcal {D}}_{\frac{1}{4},2} P|\Vert ^2_{L^{(4,2)}({\mathbb {R}})}\\&\quad \precsim \Vert (-\Delta )^\frac{1}{4} \varphi \Vert ^2_{L^{(2,\infty )}({\mathbb {R}})} \Vert |(-\Delta )^{\frac{1}{4}}P\Vert ^2_{L^{(2,2)}({\mathbb {R}})}, \end{aligned} \end{aligned}$$

where for the estimate of the last term we used again Theorem 2.2, (2.6), with $t=\frac{1}{2}$.

Combining (3.22), (3.23), and (3.24), we obtain

3.25 $$\begin{aligned} \begin{aligned} \mathcal {II} =&\int _{\mathbb {R}}\int _{\mathbb {R}}|\Omega (x,y)||d_\frac{1}{4} P(x,y)||d_\frac{1}{4} \varphi (x,y)|\frac{\,\mathrm {d}x\,\mathrm {d}y}{|x-y|}\\&\precsim \Vert \Omega \Vert _{L^2(\bigwedge \nolimits _{od}^1 {\mathbb {R}})} \Vert (-\Delta )^\frac{1}{4} \varphi \Vert _{L^{(2,\infty )}({\mathbb {R}})} [ P]_{W^{1/2,2}({\mathbb {R}})}. \end{aligned} \end{aligned}$$

Finally, from (3.16), (3.21), and (3.25) we get

$$\begin{aligned} \begin{aligned}&\Big |{\text {div}}_\frac{1}{2}(R_\varepsilon P^T{(y)})[\varphi ]\Big |\\&\quad \precsim (1+ \Vert \varepsilon \Vert _{L^\infty ({\mathbb {R}})}) \left( \Vert \Omega \Vert _{L^2(\bigwedge \nolimits _{od}^1 {\mathbb {R}})} + [P]_{W^{1/2,2}({\mathbb {R}})} \right) [P]_{W^{1/2,2}({\mathbb {R}})}\Vert (-\Delta )^{\frac{1}{4}}\varphi \Vert _{L^{(2,\infty )}({\mathbb {R}})}. \end{aligned} \end{aligned}$$

This finishes the proof of (3.14).

In order to prove (3.15) we observe

$$\begin{aligned} \left| {\text {div}}_{\frac{1}{2}} \left( (R_{\varepsilon _1} - R_{\varepsilon _2}) P^T{(y)} \right) [\varphi ] \right| \precsim \Vert \varepsilon _1 - \varepsilon _2\Vert _{L^\infty }({\mathcal {I}} + \mathcal {II}). \end{aligned}$$

Thus, in order to conclude it suffices to apply the estimates (3.21) and (3.25). $\square$

### Proof of Proposition 3.3

Let $$X = L^\infty \cap {\dot{W}}^{\frac{1}{2},2}({\mathbb {R}})$$.

For any $\varepsilon \in X$ we have $A=(1+\varepsilon )P\in L^\infty \cap {\dot{W}}^{\frac{1}{2}}({\mathbb {R}})$, which implies $A\Omega - d_\frac{1}{2} A \in L^2(\bigwedge \nolimits _{od}^1 {\mathbb {R}})$ and thus, from Lemma 3.1, we have

$$\begin{aligned} -\left( (I+\varepsilon (x,y))\, \Omega ^P(x,y) P(y) \right) {-} \left( d_{\frac{1}{2}} \varepsilon (x,y) P(y) \right) +R_\varepsilon (x,y)\in L^2(\bigwedge \nolimits _{od}^1 {\mathbb {R}}). \end{aligned}$$

We apply for this term the nonlocal Hodge decomposition, Lemma A.1: given $\varepsilon \in X$ we find $a(\varepsilon )\in W^{\frac{1}{2},2}({\mathbb {R}})$ and $B(\varepsilon )\in L^2(\bigwedge \nolimits _{od}^1{\mathbb {R}})$ with ${\text {div}}_\frac{1}{2} B(\varepsilon )=0$ satisfying

3.26 $$\begin{aligned} \begin{aligned}&- \left( (I+\varepsilon (x,y))\, \Omega ^P(x,y) P(y) \right) {-} \left( d_{\frac{1}{2}} \varepsilon (x,y) P(y) \right) +R_\varepsilon (x,y)\\&\quad = d_{\frac{1}{2}} a(\varepsilon )(x,y) + B(\varepsilon )(x,y) \end{aligned} \end{aligned}$$

with the estimates

3.27 $$\begin{aligned} \begin{aligned}&\Vert B(\varepsilon )\Vert _{L^2(\bigwedge \nolimits _{od}^1 {\mathbb {R}})} + [a(\varepsilon )]_{W^{\frac{1}{2},2}({\mathbb {R}})}\\&\quad \precsim (1+\Vert \varepsilon \Vert _{L^\infty ({\mathbb {R}})})([P]_{W^{1/2,2}({\mathbb {R}})} + \Vert \Omega \Vert _{L^2(\bigwedge \nolimits _{od}^1{\mathbb {R}})}) + [\varepsilon ]_{W^{1/2,2}({\mathbb {R}})}. \end{aligned} \end{aligned}$$

Similarly, if for any two $\varepsilon _1,\, \varepsilon _2 \in X$ we consider the difference of the corresponding Eq. (3.26) we get

3.28 $$\begin{aligned} \begin{aligned}&\Vert B(\varepsilon _1) - B(\varepsilon _2)\Vert _{L^2(\bigwedge \nolimits _{od}^1 {\mathbb {R}})}\\&\quad \precsim \Vert \varepsilon _1 - \varepsilon _2\Vert _{L^\infty ({\mathbb {R}})}([P]_{W^{1/2,2}({\mathbb {R}})} + \Vert \Omega \Vert _{L^2(\bigwedge \nolimits _{od}^1{\mathbb {R}})}) + [\varepsilon _1 - \varepsilon _2]_{W^{1/2,2}({\mathbb {R}})}. \end{aligned} \end{aligned}$$

Now we define the mapping $T:X \rightarrow X$ as the solution to

3.29 $$\begin{aligned} \begin{aligned}&- {\text {div}}_{\frac{1}{2}}\left( (I+\varepsilon (x))\, \Omega ^P(x,y) \right) {-} {\text {div}}_{\frac{1}{2}}\left( d_{\frac{1}{2}} T(\varepsilon )(x,y) \right) +{\text {div}}_{\frac{1}{2}}(R_\varepsilon (x,y) P^T(y))\\&\quad = {\text {div}}_{\frac{1}{2}} \left( B(\varepsilon )(x,y)P^T(y) \right) \end{aligned} \end{aligned}$$

with $$\lim _{|x|\rightarrow \infty }T(\varepsilon )(x)=0$$.

Using Lemma 2.1 Eq. (3.29) can be rewritten as

3.30 $$\begin{aligned} \begin{aligned} {-}(-\Delta )^{\frac{1}{2}} T(\varepsilon )&= {\text {div}}_{\frac{1}{2}} \left( B(\varepsilon )P^T{(y)} \right) + {\text {div}}_{\frac{1}{2}}\left( (I+\varepsilon {(x)})\, \Omega ^P \right) - {\text {div}}_{\frac{1}{2}}(R_\varepsilon P^T{(y)})\\&= {-B(\varepsilon )} \cdot d_\frac{1}{2} P^T + d_\frac{1}{2}(I+\varepsilon )\cdot (\Omega ^P)^*- {\text {div}}_\frac{1}{2}(R_\varepsilon P^T{(y)}){.} \end{aligned} \end{aligned}$$

We used in the second inequality Lemma 2.1.

We observe that on the right-hand we have fractional *div*-*curl*-terms: ${\text {div}}_\frac{1}{2} {B(\varepsilon )} =0$ and ${\text {div}}_\frac{1}{2} (\Omega ^P)^*=0$. Let us denote

$$\begin{aligned} \Lambda _{\varepsilon } :=(1+\Vert \varepsilon \Vert _{L^\infty ({\mathbb {R}})}) (\Vert \Omega \Vert _{L^2({\mathbb {R}})} + [P]_{W^{1/2,2}({\mathbb {R}})})[P]_{W^{1/2,2}({\mathbb {R}})}. \end{aligned}$$

By Lemma 3.4, (3.14), the rest term in (3.30) satisfies

$$\begin{aligned} \left| {\text {div}}_{\frac{1}{2}} (R_\varepsilon P^T{(y)})[\varphi ] \right| \precsim \Lambda _\varepsilon \Vert (-\Delta )^{\frac{1}{4}}\varphi \Vert _{L^{(2,\infty )}({\mathbb {R}})}. \end{aligned}$$

Thus, we may apply the nonlocal Wente’s lemma, i.e., Lemma 2.3 and obtain

3.31 $$\begin{aligned} \begin{aligned}&\Vert T(\varepsilon )\Vert _{L^\infty ({\mathbb {R}})} + [T(\varepsilon )]_{W^{1/2,2}({\mathbb {R}})}\\&\quad \precsim \Vert {B(\varepsilon )}\Vert _{L^2(\bigwedge \nolimits _{od}^1{\mathbb {R}})}[P]_{W^{1/2,2}({\mathbb {R}})} + [\varepsilon ]_{W^{1/2,2}({\mathbb {R}})}\Vert (\Omega ^P)^*\Vert _{L^2(\bigwedge \nolimits _{od}^1 {\mathbb {R}})}+ \Lambda _\varepsilon \\&\quad =\Vert B(\varepsilon )\Vert _{L^2(\bigwedge \nolimits _{od}^1{\mathbb {R}})}[P]_{W^{1/2,2}({\mathbb {R}})} + [\varepsilon ]_{W^{1/2,2}({\mathbb {R}})}\Vert \Omega ^P\Vert _{L^2(\bigwedge \nolimits _{od}^1 {\mathbb {R}})}+ \Lambda _\varepsilon . \end{aligned} \end{aligned}$$

Moreover, let $\varepsilon _1,\, \varepsilon _2 \in X$, then we have

3.32 $$\begin{aligned} \begin{aligned}&{-}(-\Delta )^{\frac{1}{2}} (T(\varepsilon _1)-T(\varepsilon _2)) \\&\quad = {\text {div}}_{\frac{1}{2}} \left( (B(\varepsilon _1) - B(\varepsilon _2))P^T(y) \right) + {\text {div}}_{\frac{1}{2}}\left( (\varepsilon _1-\varepsilon _2){(x)}\, \Omega ^P \right) - {\text {div}}_{\frac{1}{2}}((R_{\varepsilon _1}-R_{\varepsilon _2}) P^T{(y)})\\&\quad ={-(B(\varepsilon _1) - B(\varepsilon _2))}\cdot d_\frac{1}{2} P^T + d_{\frac{1}{2}}(\varepsilon _1-\varepsilon _2)\cdot (\Omega ^P)^*- {\text {div}}_{\frac{1}{2}}((R_{\varepsilon _1}-R_{\varepsilon _2}) P^T{(y)}), \end{aligned} \end{aligned}$$

where in the last equality we have used again Lemma 2.1.

Again, we observe that

$$\begin{aligned} {\text {div}}_\frac{1}{2}({B(\varepsilon _1) - (B(\varepsilon _2)}) =0 \quad \text {and} \quad {\text {div}}_\frac{1}{2} (\Omega ^P)^*=0, \end{aligned}$$

and from Lemma 3.4, (3.15), we may estimate the reminder term in (3.32)

3.33 $$\begin{aligned} |{\text {div}}_{\frac{1}{2}} (R_{\varepsilon _1} - R_{\varepsilon _2}) P^T{(y)})[\varphi ]| \precsim \Lambda _{\varepsilon _1,\varepsilon _2} \Vert (-\Delta )^{\frac{1}{4}}\varphi \Vert _{L^{(2,\infty )}({\mathbb {R}})}, \end{aligned}$$

where

3.34 $$\begin{aligned} \Lambda _{\varepsilon _1,\varepsilon _2} :=\Vert \varepsilon _1-\varepsilon _2\Vert _{L^\infty ({\mathbb {R}})} ([P]_{W^{1/2,2}({\mathbb {R}})} + \Vert \Omega \Vert _{L^2({\mathbb {R}})})[P]_{W^{1/2,2}({\mathbb {R}})}. \end{aligned}$$

Therefore, we may apply the nonlocal Wente’s Lemma 2.3 for Eq. (3.32) and obtain

3.35 $$\begin{aligned} \begin{aligned}&\Vert T(\varepsilon _1) - T(\varepsilon _2)\Vert _{L^\infty ({\mathbb {R}})} + [T(\varepsilon _1) - T(\varepsilon _2)]_{W^{1/2,2}({\mathbb {R}})}\\&\quad \precsim \Vert B(\varepsilon _1) - B(\varepsilon _2)\Vert _{L^2(\bigwedge \nolimits _{od}^1{\mathbb {R}})}[P]_{W^{1/2,2}({\mathbb {R}})} + [\varepsilon _1 - \varepsilon _2]_{W^{1/2,2}({\mathbb {R}})}\Vert \Omega ^p\Vert _{L^2(\bigwedge \nolimits _{od}^1 {\mathbb {R}})} + \Lambda _{\varepsilon _1,\varepsilon _2}. \end{aligned} \end{aligned}$$

Combining (3.35) with (3.28) and (3.34), we get

$$\begin{aligned} \begin{aligned}&\Vert T(\varepsilon _1) - T(\varepsilon _2)\Vert _{L^\infty ({\mathbb {R}})} + [T(\varepsilon _1) - T(\varepsilon _2)]_{W^{1/2,2}({\mathbb {R}})}\\&\quad \precsim \Vert \varepsilon _1-\varepsilon _2\Vert _{L^\infty ({\mathbb {R}})} \left( [P]_{W^{1/2,2}({\mathbb {R}}{)}} + \Vert \Omega \Vert _{L^2(\bigwedge \nolimits _{od}^1 {\mathbb {R}})} \right) [P]_{W^{1/2,2}({\mathbb {R}})}\\&\qquad + [\varepsilon _1 - \varepsilon _2]_{W^{1/2,2}({\mathbb {R}})}\left( [P]_{W^{1/2,2}({\mathbb {R}}{)}} + \Vert \Omega \Vert _{L^2(\bigwedge \nolimits _{od}^1 {\mathbb {R}})} \right) \\&\quad \precsim (\Vert \varepsilon _1 - \varepsilon _2\Vert _{L^\infty ({\mathbb {R}})} + [\varepsilon _1 - \varepsilon _2]_{W^{1/2,2}({\mathbb {R}})})\sigma , \end{aligned} \end{aligned}$$

where in the last inequality we used (3.11).

Thus, taking $\sigma$ small enough we obtain

$$\begin{aligned} \Vert T(\varepsilon _1) - T(\varepsilon _2)\Vert _{L^\infty ({\mathbb {R}})} + [T(\varepsilon _1) - T(\varepsilon _2)]_{W^{1/2,2}({\mathbb {R}})} \le \lambda \left( \Vert \varepsilon _1 - \varepsilon _2\Vert _{L^\infty ({\mathbb {R}})} + [\varepsilon _1 - \varepsilon _2]_{W^{1/2,2}({\mathbb {R}})} \right) , \end{aligned}$$

for a $0<\lambda <1$, which implies that *T* is a contraction. Consequently, by Banach fixed point theorem, there exists a unique $\varepsilon \in X$, such that $T(\varepsilon ) = \varepsilon$. That is we have a solution $T(\varepsilon ) = \varepsilon$, which is a solution to

$$\begin{aligned} \left\{ \begin{array}{l} -\left( (I+\varepsilon {(x)})\, \Omega ^P P{(y)} \right) {-} \left( d_{\frac{1}{2}} \varepsilon \,P{(y)} \right) +R_\varepsilon = d_{\frac{1}{2}} a(\varepsilon ) + B(\varepsilon )\\ -{\text {div}}_{\frac{1}{2}}\left( (I+\varepsilon {(x)})\, \Omega ^P \right) {-} {\text {div}}_{\frac{1}{2}}\left( d_{\frac{1}{2}} \varepsilon \right) +{\text {div}}_{\frac{1}{2}}(R_\varepsilon \,P^T{(y)}) = {\text {div}}_{\frac{1}{2}} \left( B(\varepsilon )\,P^T{(y)} \right) . \end{array} \right. \end{aligned}$$

Moreover, combining (3.31) with (3.27) and (3.11) we obtain the following estimate on $\varepsilon$

3.36 $$\begin{aligned} \Vert \varepsilon \Vert _{L^\infty ({\mathbb {R}})} + [\varepsilon ]_{W^{\frac{1}{2},2}({\mathbb {R}})} \precsim \sigma \Vert \varepsilon \Vert _{L^\infty ({\mathbb {R}})} + \sigma [\varepsilon ]_{W^{\frac{1}{2},2}({\mathbb {R}})} +\Vert \Omega \Vert _{L^2(\bigwedge \nolimits _{od}^1 {\mathbb {R}})}+[P]_{W^{\frac{1}{2},2}({\mathbb {R}})}, \end{aligned}$$

which gives for sufficiently small $\sigma$

$$\begin{aligned} \Vert \varepsilon \Vert _{L^\infty ({\mathbb {R}})} + [\varepsilon ]_{W^{\frac{1}{2},2}({\mathbb {R}})} \precsim \Vert \Omega \Vert _{L^2(\bigwedge \nolimits _{od}^1 {\mathbb {R}})}+[P]_{W^{\frac{1}{2},2}({\mathbb {R}})}. \end{aligned}$$

$$\square $$

### Proof of Theorem 1.1

By Proposition 3.3 we obtain the existence of an $\varepsilon \in L^\infty \cap {\dot{W}}^{\frac{1}{2},2}({\mathbb {R}})$, $a\in {\dot{W}}^{\frac{1}{2},2}({\mathbb {R}})$, $B\in L^2(\bigwedge \nolimits _{od}^1 {\mathbb {R}})$ with ${\text {div}}_\frac{1}{2} B =0$ satisfying the equations solution $T(\varepsilon ) = \varepsilon$, which is a solution to

$$\begin{aligned} \left\{ \begin{array}{l} -\left( (I+\varepsilon {(x)})\, \Omega ^P P{(y)} \right) {-} \left( d_{\frac{1}{2}} \varepsilon \, P{(y)} \right) +R_\varepsilon = d_{\frac{1}{2}} a + B\\ -{\text {div}}_{\frac{1}{2}}\left( (I+\varepsilon {(x)})\, \Omega ^P \right) {-} {\text {div}}_{\frac{1}{2}}\left( d_{\frac{1}{2}} \varepsilon \right) +{\text {div}}_{\frac{1}{2}}(R_\varepsilon \,P^T{(y)}) = {\text {div}}_{\frac{1}{2}} \left( BP^T{(y)} \right) , \end{array} \right. \end{aligned}$$

where $P\in {\dot{W}}^{\frac{1}{2},2}({\mathbb {R}},SO(N))$ and $\Omega ^P \in L^2(\bigwedge \nolimits _{od}^1 {\mathbb {R}})$ are taken from Theorem 2.4 and $[P]_{W^{1/2,2}({\mathbb {R}})} \precsim \Vert \Omega \Vert _{L^2(\bigwedge \nolimits _{od}^1 {\mathbb {R}})} \le \sigma$.

By Lemma 3.2 we have for sufficiently small $\sigma$

$$\begin{aligned} -\left( (I+\varepsilon {(x)})\, \Omega ^P P{(y)} \right) {-} \left( d_{\frac{1}{2}} \varepsilon \,P{(y)} \right) +R_\varepsilon = B. \end{aligned}$$

Thus, defining for $\varepsilon$ from Proposition 3.3, $A:=(I+\varepsilon ) P$, we have by Lemma 3.1

$$\begin{aligned} A\Omega - d_\frac{1}{2} A = B. \end{aligned}$$

The invertibility of *A* follows from the invertibility of *P* and $I+\varepsilon$. Finally, since $A = (I+\varepsilon )P$, we obtain from (3.13) and (2.8) the estimates

$$\begin{aligned}{}[A]_{W^{\frac{1}{2},2}({\mathbb {R}})} \precsim (1+\Vert \varepsilon \Vert _{L^\infty }) [P]_{W^{\frac{1}{2},2}({\mathbb {R}})} + [\varepsilon ]_{W^{\frac{1}{2},2}({\mathbb {R}})} \precsim \Vert \Omega \Vert _{L^2(\bigwedge \nolimits _{od}^1 {\mathbb {R}})}, \end{aligned}$$

and

$$\begin{aligned} \Vert A\Vert _{L^\infty ({\mathbb {R}})} \precsim 1 + \Vert \Omega \Vert _{L^2(\bigwedge \nolimits _{od}^1 {\mathbb {R}})}. \end{aligned}$$

This finishes the proof. $\square$

## Weak convergence result: Proof of Theorem 1.3

Using Lemma 2.1, we obtain the following.

### Lemma 4.1

Assume that $\Omega \in L^2(\bigwedge \nolimits _{od}^1 {\mathbb {R}})$. Then $u\in {\dot{W}}^{\frac{1}{2},2}({\mathbb {R}},{\mathbb {R}}^N)\cap (L^2+L^\infty ({\mathbb {R}}))$ is a solution to

4.1 $$\begin{aligned} (-\Delta )^{\frac{1}{2}}u^i = \Omega \cdot d_{\frac{1}{2}} u \end{aligned}$$

if and only if for any invertible matrix-valued function $A,A^{-1}\in L^\infty \cap {\dot{W}}^{\frac{1}{2},2}({\mathbb {R}},GL(N))$,

$$\begin{aligned} {\text {div}}_{\frac{1}{2}} (A_{ik} d_{\frac{1}{2}} u^k) =\left( A_{ij}\Omega _{j k}\, -d_{\frac{1}{2}} A_{ik} \right) \cdot d_{\frac{1}{2}}u^k. \end{aligned}$$

In a first step we prove the “local version” of Theorem 1.3.

### Proposition 4.2

Let $\sigma > 0$ be the number from Theorem 1.1. Let $\{u_\ell \}_{\ell \in {{\mathbb {N}}}}$ be a sequence as in Theorem 1.3 of solutions to

$$\begin{aligned} (-\Delta )^{\frac{1}{2}} u_\ell = \Omega _{\ell } \cdot d_\frac{1}{2} u_\ell + f_\ell \quad \text {in } {\mathcal {D}}'({\mathbb {R}}). \end{aligned}$$

Additionally, let us assume that for some bounded interval $D \subset {\mathbb {R}}$ we have

4.2 $$\begin{aligned} \sup _{\ell } \Vert \Omega _\ell \Vert _{L^2(\bigwedge \nolimits ^1_{od} D)} < \sigma . \end{aligned}$$

Then

$$\begin{aligned} (-\Delta )^{\frac{1}{2}} u = \Omega \cdot d_\frac{1}{2} u +f\quad \text {in } {\mathcal {D}}'(D). \end{aligned}$$

### Proof

Let us define $\Omega _{D,\ell } :=\chi _{D}(x) \chi _{D}(y) \Omega _\ell \in L^2(\bigwedge \nolimits _{od}^1 {\mathbb {R}})$. Then by (4.2) we have

4.3 $$\begin{aligned} \Vert \Omega _{D,\ell }\Vert _{L^2(\bigwedge \nolimits _{od}^1 {\mathbb {R}})} \le \Vert \Omega _\ell \Vert _{L^2(\bigwedge \nolimits _{od}^1 D)} < \sigma . \end{aligned}$$

By Theorem 1.1 for $\Omega _{D,\ell }$ there exists a gauge $A_\ell$ such that

4.4 $$\begin{aligned} {\text {div}}_\frac{1}{2}(\Omega _{D,\ell }^{A_\ell }) =0, \end{aligned}$$

where $$\Omega _{D,\ell }^{A_\ell }:=A_\ell \Omega _{D,\ell } - d_\frac{1}{2} A_\ell $$.

Let $D_1 \subset \subset D$ be an open set.

By assumption and Lemma 4.1, we have for any $\psi \in C_c^\infty (D_1)$ and for $\Omega _\ell ^{A_\ell }=A_\ell \Omega _\ell - d_{\frac{1}{2}}A_\ell$

$$\begin{aligned} \int _{{\mathbb {R}}} A_\ell d_\frac{1}{2} u_\ell \cdot d_\frac{1}{2} \psi = \int _{{\mathbb {R}}} \Omega _{\ell }^{ A_\ell } {\cdot \,} d_{\frac{1}{2}} u_\ell \, \psi + f_\ell [ A_\ell \psi ]. \end{aligned}$$

Here with a slight abuse of notation we write for the matrix product $\left( f[A\psi ] \right) ^i :=\sum _{k} f^k[A^{ik} \psi ]$.

Let us denote $\Omega _{D^c,\ell } :=\Omega _\ell - \Omega _{D,\ell }$. Then we have

$$\begin{aligned} \int _{{\mathbb {R}}} A_\ell d_\frac{1}{2} u_\ell \cdot d_\frac{1}{2} \psi = \int _{{\mathbb {R}}} \Omega _{D,\ell }^{A_\ell }\cdot d_{\frac{1}{2}} u_\ell \, \psi +\int _{{\mathbb {R}}} A_\ell \Omega _{D^c,\ell }\cdot d_{\frac{1}{2}} u_\ell \, \psi + f_\ell [A_\ell \psi ]. \end{aligned}$$

By Lemma 2.1 and (4.4), we have ${\text {div}}_\frac{1}{2} \left( \left( \Omega _{D,\ell }^{A_\ell } \right) ^* \right) =0$, thus again by Lemma 2.1 we get $\Omega _{D,\ell }^{A_\ell }\cdot d_{\frac{1}{2}} u_\ell = {\text {div}}_{\frac{1}{2}}\left( \left( \Omega _{D,\ell }^{A_\ell } \right) ^*u_\ell (x) \right)$. Therefore,

4.5 $$\begin{aligned} \int _{{\mathbb {R}}} A_\ell d_\frac{1}{2} u_\ell \cdot d_\frac{1}{2} \psi = \int _{{\mathbb {R}}} \left( \Omega _{D,\ell }^{A_\ell } \right) ^*\cdot u_\ell d_{\frac{1}{2}}\psi +\int _{{\mathbb {R}}} A_\ell \Omega _{D^c,\ell }\cdot d_{\frac{1}{2}} u_\ell \, \psi + f_\ell [A_\ell \psi ]. \end{aligned}$$

We will pass with $\ell \rightarrow \infty$ in (4.5). Roughly speaking, the convergence of most of the terms will be a result of a combination of weak–strong convergence. We first observe that by Theorem 1.1 we have

$$\begin{aligned} \Vert A_\ell \Vert _{{\dot{W}}^{\frac{1}{2},2}({\mathbb {R}})} \precsim \Vert \Omega _{D,\ell }\Vert _{L^2(\bigwedge \nolimits _{od}^1 {\mathbb {R}})} \le \sigma \quad \text { and } \quad \Vert A_\ell \Vert _{L^\infty ({\mathbb {R}})} \precsim 1+\sigma . \end{aligned}$$

Thus, $\sup _\ell \Vert A_\ell \Vert _{{\dot{W}}^{\frac{1}{2},2}({\mathbb {R}})} <\infty$ and $\sup _\ell \Vert A_\ell \Vert _{L^\infty }({\mathbb {R}}) < \infty$. Up to taking a subsequence we obtain

4.6 $$\begin{aligned} A_\ell \rightharpoonup A \quad \text { weakly in }{\dot{W}}^{\frac{1}{2},2}({\mathbb {R}},{\mathbb {R}}^N), \quad A_\ell \rightarrow A \quad \text { locally strongly in }L^2, \end{aligned}$$

where we used the Rellich–Kondrachov’s compact embedding theorem and $A\in L^\infty \cap {\dot{W}}^{\frac{1}{2},2}({\mathbb {R}},GL(N))$. By the pointwise a.e. convergence, we have $\Vert A\Vert _{L^\infty ({\mathbb {R}})}\precsim 1+ \sigma$.

By (4.3) we also have up to a subsequence

$$\begin{aligned} \Omega _{D,\ell } \rightharpoonup \Omega _D \quad \text { weakly in }L^2\left(\bigwedge \nolimits _{od}^1 {\mathbb {R}}\right), \end{aligned}$$

where $$\Omega _D\in L^2(\bigwedge \nolimits _{od}^1 {\mathbb {R}})$$.

By assumptions of the Theorem we also have, up to a subsequence,

$$\begin{aligned} u_\ell \rightharpoonup u \quad \text { weakly in } {\dot{W}}^{\frac{1}{2},2}({\mathbb {R}}), \quad u_\ell \rightarrow u \quad \text { locally strongly in }L^2, \end{aligned}$$

where $$u\in {\dot{W}}^{\frac{1}{2},2}({\mathbb {R}},{\mathbb {R}}^N)$$.

Let us choose a large $R \gg 1$, such that in particular $D_1 \subset B(R)$. We begin with the first term of (4.5).

*Step 1.* We claim that (up to a subsequence)

4.7 $$\begin{aligned} \lim _{\ell \rightarrow \infty } \int _{{\mathbb {R}}} A_\ell d_\frac{1}{2} u_\ell \cdot d_\frac{1}{2} \psi = \int _{{\mathbb {R}}} A d_\frac{1}{2} u \cdot d_\frac{1}{2} \psi . \end{aligned}$$

Indeed, we observe

4.8 $$\begin{aligned} \begin{aligned} \int _{{\mathbb {R}}} A_\ell d_\frac{1}{2} u_\ell \cdot d_\frac{1}{2} \psi - \int _{{\mathbb {R}}} A d_\frac{1}{2} u \cdot d_\frac{1}{2} \psi&= \int _{{\mathbb {R}}} (A_\ell - A)d_\frac{1}{2} u_\ell \cdot d_\frac{1}{2} \psi \\&\quad + \int _{\mathbb {R}}A (d_\frac{1}{2} u_\ell - d_\frac{1}{2} u) \cdot d_\frac{1}{2} \psi . \end{aligned} \end{aligned}$$

By weak convergence of $d_\frac{1}{2} u_\ell$ in $L^2(\bigwedge \nolimits _{od}^1 {\mathbb {R}})$, we have

4.9 $$\begin{aligned} \lim _{\ell \rightarrow \infty } \int _{\mathbb {R}}A (d_\frac{1}{2} u_\ell - d_\frac{1}{2} u) \cdot d_\frac{1}{2} \psi =0. \end{aligned}$$

As for the first term on the right-hand side of (4.8), we observe that since $\mathrm{supp\,}\psi \subset D_1\subset B(R)$,

4.10 $$\begin{aligned} \begin{aligned}&\int _{{\mathbb {R}}}\int _{{\mathbb {R}}} (A_\ell (x) - A(x)) \frac{(u_\ell (x)-u_\ell (y)) (\psi (x)-\psi (y))}{|x-y|^{2}}\,\mathrm {d}x\,\mathrm {d}y\\&\quad = \int _{B(R)}\int _{B(R)} (A_\ell (x) - A(x)) \frac{(u_\ell (x)-u_\ell (y)) (\psi (x)-\psi (y))}{|x-y|^{2}}\,\mathrm {d}x\,\mathrm {d}y\\&\qquad + \int _{{\mathbb {R}}}\int _{B(R)} (A_\ell (x) - A(x)) \frac{(u_\ell (x)-u_\ell (y)) (\psi (x)-\psi (y))}{|x-y|^{2}}\,\mathrm {d}x\,\mathrm {d}y\\&\qquad +\int _{B(R)}\int _{{\mathbb {R}}\setminus B(R)} (A_\ell (x) - A(x)) \frac{(u_\ell (x)-u_\ell (y)) (\psi (x)-\psi (y))}{|x-y|^{2}}\,\mathrm {d}x\,\mathrm {d}y. \end{aligned} \end{aligned}$$

By strong convergence in $L^2$ of $A_\ell$ on compact domains, we have

4.11 $$\begin{aligned} \begin{aligned}&\lim _{\ell \rightarrow \infty } \int _{B(R)}\int _{B(R)} (A_\ell (x) - A(x)) \frac{(u_\ell (x)-u_\ell (y)) (\psi (x)-\psi (y))}{|x-y|^{2}}\,\mathrm {d}x\,\mathrm {d}y\\&\quad \precsim \lim _{\ell \rightarrow \infty } \Vert A_\ell - A\Vert _{L^2(B(R))}\Vert \psi \Vert _{\mathrm{Lip\,}}[u_\ell ]_{W^{\frac{1}{2},2}(B(R))} =0 \end{aligned} \end{aligned}$$

and (noting once again that $\mathrm{supp\,}\psi \subset D_1$)

4.12 $$\begin{aligned} \begin{aligned}&\lim _{\ell \rightarrow \infty } \left| \int _{{\mathbb {R}}\setminus B(R)}\int _{B(R)} (A_\ell (x) - A(x)) \frac{(u_\ell (x)-u_\ell (y))(\psi (x)-\psi (y))}{|x-y|^{2}}\,\mathrm {d}x\,\mathrm {d}y \right| \\&\quad \precsim \lim _{\ell \rightarrow \infty }{\Vert A_\ell -A\Vert _{L^2(B(R))} [u_\ell ]_{W^{\frac{1}{2},2}({\mathbb {R}})} \left( \int _{{\mathbb {R}}\setminus B(R)} \sup _{x\in D_1} \frac{|\psi (x) - \psi (y)|^2}{|x-y|^2} \,\mathrm {d}y \right) ^\frac{1}{2}}\\&\quad {\precsim \lim _{\ell \rightarrow \infty }\Vert A_\ell -A\Vert _{L^2(B(R))} [u_\ell ]_{W^{\frac{1}{2},2}({\mathbb {R}})} \Vert \psi \Vert _{L^\infty } \left( \int _{{\mathbb {R}}\setminus B(R)} \frac{1}{1+|y|^2} \,\mathrm {d}y \right) ^\frac{1}{2}} =0. \end{aligned} \end{aligned}$$

In the last inequality we used the fact that if $x\in D_1$ and $y\in {\mathbb {R}}\setminus B(R)$ then $|x-y|\succsim 1 + |y|$.

For the last term of (4.10), we similarly use that if $y \in \mathrm{supp\,}\psi$ and $x \in {\mathbb {R}}\setminus B(R)$, then we have $|x-y| \succsim 1+|x|$ with a constant independent of *R*.

$$\begin{aligned} \begin{aligned}&\left| \int _{B(R)}\int _{{\mathbb {R}}\setminus B(R)} (A_\ell (x) - A(x)) \frac{(u_\ell (x)-u_\ell (y)) (\psi (x)-\psi (y))}{|x-y|^{2}}\,\mathrm {d}x\,\mathrm {d}y \right| \\&\quad \precsim \left( \Vert A_\ell \Vert _{L^\infty } + \Vert A\Vert _{L^\infty } \right) \Vert \psi \Vert _{L^\infty } \int _{D_1}\int _{{\mathbb {R}}\setminus B(R)} \frac{|u_\ell (x)|+|u_\ell (y)|}{1+|x|^{2}}\,\mathrm {d}x\,\mathrm {d}y\\&\quad \precsim \left( \Vert A_\ell \Vert _{L^\infty } + \Vert A\Vert _{L^\infty } \right) \Vert \psi \Vert _{L^\infty }\, \left( \Vert u_\ell \Vert _{L^2(D_1)} \int _{{\mathbb {R}}\setminus B(R)} \frac{1}{1+|x|^2} \,\mathrm {d}x \right) \\&\qquad + \left( \Vert A_\ell \Vert _{L^\infty } + \Vert A\Vert _{L^\infty } \right) \Vert \psi \Vert _{L^\infty }\,\\&\qquad \left( \Vert u_\ell \Vert _{L^\infty +L^2({\mathbb {R}})} \left( \int _{{\mathbb {R}}\setminus B(R)} \frac{\,\mathrm {d}x}{1+|x|^2} + \left( \int _{{\mathbb {R}}\setminus B(R)} \frac{\,\mathrm {d}x}{(1+|x|^2)^2} \right) ^{\frac{1}{2}} \right) \right) \\&\quad \precsim \left( \Vert A_\ell \Vert _{L^\infty } + \Vert A\Vert _{L^\infty } \right) \Vert \psi \Vert _{L^\infty }\, \left( \Vert u\Vert _{L^2(D_1)} + \Vert u_\ell \Vert _{L^\infty +L^2({\mathbb {R}})} \right) R^{-\frac{1}{2}}. \end{aligned} \end{aligned}$$

So we have

4.13 $$\begin{aligned} \lim _{R \rightarrow \infty } \sup _{\ell } \left| \int _{B(R)}\int _{{\mathbb {R}}\setminus B(R)} (A_\ell (x) - A(x)) \frac{(u_\ell (x)-u_\ell (y)) (\psi (x)-\psi (y))}{|x-y|^{2}}\,\mathrm {d}x\,\mathrm {d}y \right| = 0 {.} \end{aligned}$$

By (4.10), (4.11), (4.12), and (4.13) we obtain the convergence of the first term on the right-hand side of (4.8), i.e.,

4.14 $$\begin{aligned} \lim _{\ell \rightarrow \infty } \int _{{\mathbb {R}}}\int _{{\mathbb {R}}} (A_\ell (x) - A(x)) \frac{(u_\ell (x)-u_\ell (y)) (\psi (x)-\psi (y))}{|x-y|^{2}}\,\mathrm {d}x\,\mathrm {d}y= 0. \end{aligned}$$

Thus, combining (4.8), (4.9), and (4.14) we obtain the claim (4.7).

*Step  2.* We claim that (up to a subsequence)

4.15 $$\begin{aligned} \lim _{\ell \rightarrow \infty }\int _{{\mathbb {R}}} \left( \Omega _{D,\ell }^{A_\ell } \right) ^*\cdot u_\ell d_{\frac{1}{2}}\psi = \int _{{\mathbb {R}}} \left( \Omega _{D}^{A} \right) ^*\cdot u d_{\frac{1}{2}}\psi , \end{aligned}$$

where $$\Omega _D^A :=A\Omega _D - d_\frac{1}{2} A$$.

Indeed, we write

4.16 $$\begin{aligned} \begin{aligned}&\int _{{\mathbb {R}}} \left( \Omega _{D,\ell }^{A_\ell } \right) ^*\cdot u_\ell d_{\frac{1}{2}}\psi -\int _{{\mathbb {R}}} \left( \Omega _{D}^{A} \right) ^*\cdot u d_{\frac{1}{2}}\psi \\&\quad = \int _{\mathbb {R}}\int _{\mathbb {R}}\left( \left( \Omega _{D,\ell }^{A_\ell } \right) ^*(x,y) u_\ell (x) - \left( \Omega _{D}^{A} \right) ^*(x,y)u(x) \right) \frac{\psi (x)-\psi (y)}{|x-y|^\frac{1}{2}}\frac{\,\mathrm {d}x \,\mathrm {d}y}{|x-y|}\\&\quad = {\int _{\mathbb {R}}\int _{\mathbb {R}}\left( A_\ell (y)\Omega _{D,\ell }(y,x)u_\ell (x) - A(y)\Omega _D(y,x)u(x) \right) \frac{\psi (x)-\psi (y)}{|x-y|^\frac{1}{2}}\frac{\,\mathrm {d}x \,\mathrm {d}y}{|x-y|}}\\&\qquad {- \int _{\mathbb {R}}\int _{\mathbb {R}}\left( d_\frac{1}{2} A_\ell (y,x)u_\ell (x) - d_\frac{1}{2} A(y,x)u(x) \right) \frac{\psi (x)-\psi (y)}{|x-y|^\frac{1}{2}}\frac{\,\mathrm {d}x \,\mathrm {d}y}{|x-y|}}. \end{aligned} \end{aligned}$$

Now, in order to obtain

4.17 $$\begin{aligned} \lim _{\ell \rightarrow 0}\int _{\mathbb {R}}\int _{\mathbb {R}}\left( A_\ell (y)\Omega _{D,\ell }(y,x)u_\ell (x) - A(y)\Omega _D(y,x)u(x) \right) \frac{\psi (x)-\psi (y)}{|x-y|^\frac{1}{2}}\frac{\,\mathrm {d}x \,\mathrm {d}y}{|x-y|}=0 \end{aligned}$$

we split the integral in two

4.18 $$\begin{aligned} \begin{aligned}&{\int _{\mathbb {R}}\int _{\mathbb {R}}\left( A_\ell (y)\Omega _{D,\ell }(y,x)u_\ell (x) - A(y)\Omega _D(y,x)u(x) \right) \frac{\psi (x)-\psi (y)}{|x-y|^\frac{1}{2}}\frac{\,\mathrm {d}x \,\mathrm {d}y}{|x-y|}}\\&\quad {= \int _{\mathbb {R}}\int _{\mathbb {R}}\left( A(y)u(x)(\Omega _{D,\ell }(y,x)-\Omega _D(y,x)) \right) \frac{\psi (x)-\psi (y)}{|x-y|^\frac{1}{2}}\frac{\,\mathrm {d}x \,\mathrm {d}y}{|x-y|}}\\&\qquad {+ \int _{\mathbb {R}}\int _{\mathbb {R}}\left( A_\ell (y)u_\ell (x) - A(y)u(x) \right) \Omega _{D,\ell }(y,x) \frac{\psi (x)-\psi (y)}{|x-y|^\frac{1}{2}}\frac{\,\mathrm {d}x \,\mathrm {d}y}{|x-y|}.} \end{aligned} \end{aligned}$$

The first term on the right-hand side of (4.18) converges to zero as $\ell \rightarrow \infty$. This follows from the weak convergence of $\Omega _{D,\ell } \rightharpoonup \Omega _D$ in $L^2(\bigwedge \nolimits ^1_{od}{\mathbb {R}})$, the fact that $\Omega _{D,\ell } - \Omega _D$ is supported on $D\times D$, and that $A(y)u(x)d_\frac{1}{2}\psi (x,y)\chi _{D}(x) \chi _{D}(y) \in L^2(\bigwedge \nolimits ^1_{od}{\mathbb {R}})$ (the easy verification of the latter is left to the reader).

As for the second term on the right-hand side of (4.18), we begin with the observation that

4.19 $$\begin{aligned} \begin{aligned}&{\int _{\mathbb {R}}\int _{\mathbb {R}}\left( A_\ell (y)u_\ell (x) - A(y)u(x) \right) \Omega _{D,\ell }(y,x) \frac{\psi (x)-\psi (y)}{|x-y|^\frac{1}{2}}\frac{\,\mathrm {d}x \,\mathrm {d}y}{|x-y|}}\\&\quad {= \int _{\mathbb {R}}\int _{\mathbb {R}}\left( A_\ell (y) - A(y) \right) u_\ell (x)\Omega _{D,\ell }(y,x) \frac{\psi (x)-\psi (y)}{|x-y|^\frac{1}{2}}\frac{\,\mathrm {d}x \,\mathrm {d}y}{|x-y|}}\\&\qquad {+ \int _{\mathbb {R}}\int _{\mathbb {R}}A(y)(u_\ell (x) - u(x))\Omega _{D,\ell }(y,x) \frac{\psi (x)-\psi (y)}{|x-y|^\frac{1}{2}}\frac{\,\mathrm {d}x \,\mathrm {d}y}{|x-y|}.} \end{aligned} \end{aligned}$$

To estimate the first term of the right-hand side of (4.19), we first note that the support of $\Omega _{D,\ell }$ is $D\times D$ and then we use Hölder’s inequality

4.20 $$\begin{aligned} \begin{aligned}&{\lim _{\ell \rightarrow \infty }\left| \int _{{\mathbb {R}}} \int _{{\mathbb {R}}}(A_\ell (y) - A(y))u_\ell (x)\Omega _{D,\ell }(y,x) \frac{\psi (x)-\psi (y)}{|x-y|^\frac{1}{2}}\frac{\,\mathrm {d}x \,\mathrm {d}y}{|x-y|} \right| }\\&\quad {\le \lim _{\ell \rightarrow \infty }\int _{D} \int _{D}\left| A_\ell (y) - A(y) \right| \left| u_\ell (x) \right| \left| \Omega _{D,\ell }(y,x) \right| \frac{\left| \psi (x)-\psi (y) \right| }{|x-y|^\frac{1}{2}}\frac{\,\mathrm {d}x \,\mathrm {d}y}{|x-y|}}\\&\quad {\le \lim _{\ell \rightarrow \infty } \Vert A_\ell - A\Vert _{L^2(D)} \Vert u_\ell \Vert _{L^2(D)}\Vert \Omega _{D,\ell }\Vert _{L^2(\bigwedge \nolimits ^1_{od}{\mathbb {R}})} \Vert \psi \Vert _{\mathrm{Lip\,}}=0.} \end{aligned} \end{aligned}$$

Now we verify the convergence of the second term of the right-hand side of (4.19). Again we use that the support of $\Omega _{D,\ell }$ is $D\times D$ and thus by the strong convergence in $L^2$ of $u_\ell$ on compact domains we have

4.21 $$\begin{aligned} \begin{aligned}&{\lim _{\ell \rightarrow \infty }\left| \int _{\mathbb {R}}\int _{\mathbb {R}}A(y)(u_\ell (x) - u(x))\Omega _{D,\ell }(y,x) \frac{\psi (x)-\psi (y)}{|x-y|^\frac{1}{2}}\frac{\,\mathrm {d}x \,\mathrm {d}y}{|x-y|} \right| }\\&\quad {\le \lim _{\ell \rightarrow \infty }\int _{D}\int _{D}\left| A(y)(u_\ell (x) - u(x))\Omega _{D,\ell }(y,x) \right| \frac{\left| \psi (x)-\psi (y) \right| }{|x-y|^\frac{1}{2}}\frac{\,\mathrm {d}x \,\mathrm {d}y}{|x-y|}}\\&\quad {\precsim \lim _{\ell \rightarrow \infty } \Vert A\Vert _{L^\infty }\Vert u_\ell - u\Vert _{L^2(D)} \Vert \Omega _{D,\ell }\Vert _{L^2(\bigwedge \nolimits _{od}^1 {\mathbb {R}})}\Vert \psi \Vert _{\mathrm{Lip\,}}=0.} \end{aligned} \end{aligned}$$

We also claim that

4.22 $$\begin{aligned} \lim _{\ell \rightarrow \infty } \int _{\mathbb {R}}\int _{\mathbb {R}}\left( d_\frac{1}{2} A_\ell (y,x)u_\ell (x) - d_\frac{1}{2} A(y,x)u(x) \right) \frac{\psi (x)-\psi (y)}{|x-y|^\frac{1}{2}}\frac{\,\mathrm {d}x \,\mathrm {d}y}{|x-y|} =0. \end{aligned}$$

To verify this statement, we divide the integral in two

4.23 $$\begin{aligned} \begin{aligned}&{\int _{\mathbb {R}}\int _{\mathbb {R}}\left( d_\frac{1}{2} A_\ell (y,x)u_\ell (x) - d_\frac{1}{2} A(y,x)u(x) \right) \frac{\psi (x)-\psi (y)}{|x-y|^\frac{1}{2}}\frac{\,\mathrm {d}x \,\mathrm {d}y}{|x-y|}}\\&\quad {= \int _{\mathbb {R}}\int _{\mathbb {R}}d_\frac{1}{2} A_\ell (y,x)(u_\ell (x) - u(x))\frac{\psi (x)-\psi (y)}{|x-y|^\frac{1}{2}}\frac{\,\mathrm {d}x \,\mathrm {d}y}{|x-y|}} \\&\qquad {+ \int _{\mathbb {R}}\int _{\mathbb {R}}(d_\frac{1}{2} A_\ell (y,x) - d_\frac{1}{2}A(y,x))u(x)\frac{\psi (x)-\psi (y)}{|x-y|^\frac{1}{2}}\frac{\,\mathrm {d}x \,\mathrm {d}y}{|x-y|}.} \end{aligned} \end{aligned}$$

The second term on the right-hand side of (4.23) converges to zero as $\ell \rightarrow \infty$, because $d_\frac{1}{2} A\rightharpoonup d_\frac{1}{2} A$ weakly in $L^2(\bigwedge \nolimits ^1_{od} {\mathbb {R}})$ and $u(x)d_\frac{1}{2} \psi (x,y) \in L^2(\bigwedge \nolimits ^1_{od}{\mathbb {R}})$.

We verify the convergence of the first term on the right-hand side of (4.23). First we note that by the strong convergence of $u_\ell$ in $L^2$ on compact domains we have

4.24 $$\begin{aligned} \begin{aligned}&{\lim _{\ell \rightarrow \infty }\int _{B(R)}\int _{B(R)} d_\frac{1}{2} A_\ell (y,x)(u_\ell (x) - u(x))\frac{\psi (x)-\psi (y)}{|x-y|^\frac{1}{2}}\frac{\,\mathrm {d}x \,\mathrm {d}y}{|x-y|}}\\&\quad {\le \Vert u_\ell - u\Vert _{L^2(B(R))} \Vert \psi \Vert _{\mathrm{Lip\,}}[A_\ell ]_{W^{\frac{1}{2},2}({\mathbb {R}})} =0} \end{aligned} \end{aligned}$$

and

4.25 $$\begin{aligned} \begin{aligned}&{\lim _{\ell \rightarrow \infty }\int _{{\mathbb {R}}\setminus B(R)} \int _{B(R)}d_\frac{1}{2} A_\ell (y,x)(u_\ell (x) - u(x))\frac{\psi (x)-\psi (y)}{|x-y|^\frac{1}{2}}\frac{\,\mathrm {d}x \,\mathrm {d}y}{|x-y|}}\\&\quad {\precsim \lim _{\ell \rightarrow \infty }\Vert u_\ell - u\Vert _{L^2(B(R))}[A_\ell ]_{W^{\frac{1}{2},2}({\mathbb {R}})}\Vert \psi \Vert _{L^\infty }\left( \int _{{\mathbb {R}}\setminus B(R)} \frac{1}{1+|y|^2} \,\mathrm {d}y \right) ^\frac{1}{2} =0.} \end{aligned} \end{aligned}$$

Finally, we have since $\mathrm{supp\,}\psi \subset D_1 \subset B(R)$

4.26 $$\begin{aligned} \begin{aligned}&{\int _{B(R)} \int _{{\mathbb {R}}\setminus B(R)}d_\frac{1}{2} A_\ell (y,x)(u_\ell (x) - u(x))\frac{\psi (x)-\psi (y)}{|x-y|^\frac{1}{2}}\frac{\,\mathrm {d}x \,\mathrm {d}y}{|x-y|}}\\&\quad {\precsim [A_\ell ]_{W^{\frac{1}{2},2}({\mathbb {R}})} \Vert \psi \Vert _{L^\infty }\left( \int _{{\mathbb {R}}\setminus B(R)}\frac{|u_\ell (x) - u(x)|^2}{1+|x|^2}\,\mathrm {d}x \right) ^\frac{1}{2}}\\&\quad {\precsim [A_\ell ]_{W^{\frac{1}{2},2}({\mathbb {R}})}\Vert \psi \Vert _{L^\infty }\Vert u_\ell - u\Vert _{L^2+L^\infty ({\mathbb {R}})}\max \left\{ \left( \int _{{\mathbb {R}}\setminus B(R)} \frac{1}{1+|x|^2}\,\mathrm {d}x \right) ^\frac{1}{2}, \left( \frac{1}{1+R^2} \right) ^\frac{1}{2}\right\} }\\&\quad {\precsim R^{-\frac{1}{2}}[A_\ell ]_{W^{\frac{1}{2},2}({\mathbb {R}})} \Vert \psi \Vert _{L^\infty }\Vert u_\ell - u\Vert _{L^2+L^\infty ({\mathbb {R}})}.} \end{aligned} \end{aligned}$$

This gives

4.27 $$\begin{aligned} \lim _{R\rightarrow \infty } \sup _\ell \left| \int _{B(R)} \int _{{\mathbb {R}}\setminus B(R)}d_\frac{1}{2} A_\ell (y,x)(u_\ell (x) - u(x))\frac{\psi (x)-\psi (y)}{|x-y|^\frac{1}{2}}\frac{\,\mathrm {d}x \,\mathrm {d}y}{|x-y|} \right| =0. \end{aligned}$$

Thus, the convergence of the first term of (4.23) follows from (4.24), (4.25), and (4.27). We proved (4.22).

Now (4.15) follows from (4.16) combined with (4.17) and (4.22).

*Step  3.* We claim that

4.28 $$\begin{aligned} {\text {div}}_\frac{1}{2} \left( \Omega _{D}^A \right) ^*=0. \end{aligned}$$

That is, we claim that for any $\varphi \in C_c^\infty ({\mathbb {R}})$ we have

$$\begin{aligned} 0=\lim _{\ell \rightarrow \infty } \int _{{\mathbb {R}}} \int _{{\mathbb {R}}} \left( \Omega _{D,\ell }^{A_\ell } \right) ^*\frac{\varphi (x) - \varphi (y)}{|x-y|^\frac{1}{2}} \frac{\,\mathrm {d}x \,\mathrm {d}y}{|x-y|} = \int _{{\mathbb {R}}} \int _{{\mathbb {R}}} \left( \Omega _{D}^{A} \right) ^*\frac{\varphi (x) - \varphi (y)}{|x-y|^\frac{1}{2}} \frac{\,\mathrm {d}x \,\mathrm {d}y}{|x-y|}. \end{aligned}$$

We write

4.29 $$\begin{aligned} \begin{aligned}&\int _{{\mathbb {R}}} \int _{{\mathbb {R}}} \left( \Omega _{D,\ell }^{A_\ell } \right) ^*\frac{\varphi (x) - \varphi (y)}{|x-y|^\frac{1}{2}} \frac{\,\mathrm {d}x \,\mathrm {d}y}{|x-y|} - \int _{{\mathbb {R}}} \int _{{\mathbb {R}}} \left( \Omega _{D}^{A} \right) ^*\frac{\varphi (x) - \varphi (y)}{|x-y|^\frac{1}{2}} \frac{\,\mathrm {d}x \,\mathrm {d}y}{|x-y|}\\&\quad = \int _{\mathbb {R}}\int _{\mathbb {R}}\left( A(y)\Omega _D(y,x) - A_\ell (y)\Omega _{D,\ell }(y,x) \right) d_\frac{1}{2} \varphi (x,y) \frac{\,\mathrm {d}x \,\mathrm {d}y}{|x-y|}\\&\qquad + \int _{\mathbb {R}}\int _{\mathbb {R}}\left( d_\frac{1}{2} A(y,x) - d_\frac{1}{2} A_\ell (y,x) \right) d_\frac{1}{2} \varphi (x,y) \frac{\,\mathrm {d}x \,\mathrm {d}y}{|x-y|}. \end{aligned} \end{aligned}$$

As for the second term of (4.29), we observe that by weak convergence of $d_\frac{1}{2} A_\ell$ in $L^2(\bigwedge \nolimits _{od}^1 {\mathbb {R}})$ we have

$$\begin{aligned} \lim _{\ell \rightarrow \infty } \int _{\mathbb {R}}\int _{\mathbb {R}}\left( d_\frac{1}{2} A(y,x) - d_\frac{1}{2} A_\ell (y,x) \right) d_\frac{1}{2} \varphi (x,y) \frac{\,\mathrm {d}x \,\mathrm {d}y}{|x-y|} =0. \end{aligned}$$

As for the first term of (4.29), we proceed exactly as in Step 1 and obtain

$$\begin{aligned} \lim _{\ell \rightarrow \infty } \int _{\mathbb {R}}\int _{\mathbb {R}}\left( A(y)\Omega _D(y,x) - A_\ell (y)\Omega _{D,\ell }(y,x) \right) d_\frac{1}{2} \varphi (x,y) \frac{\,\mathrm {d}x \,\mathrm {d}y}{|x-y|} =0. \end{aligned}$$

This finishes the proof of (4.28).

*Step  4.* We claim that (up to a subsequence)

4.30 $$\begin{aligned} \lim _{\ell \rightarrow \infty } A_\ell \Omega _{D^c,\ell } \cdot d_\frac{1}{2} u_\ell \psi = \int _{\mathbb {R}}A\Omega _{D^c} \cdot d_\frac{1}{2} u\psi , \end{aligned}$$

where $\Omega _{D^c} = \Omega - \Omega _D$ and $\Omega \in L^2(\bigwedge \nolimits _{od}^1 {\mathbb {R}})$ is the one given in the assumptions of the theorem.

Indeed, since $\Omega _{D^c,\ell }(x,y)=0$ whenever both $x,y \in D$ we have by the support of $\psi$,

4.31 $$\begin{aligned} \begin{aligned}&\int _{{\mathbb {R}}} A_\ell \Omega _{D^c,\ell }\cdot d_{\frac{1}{2}} u \psi \\&\quad = \int _{{\mathbb {R}}} \int _{{\mathbb {R}}} (A_\ell (x))_{ij} (\Omega _{D^c,\ell })_{jk}(x,y) \frac{\left( u_\ell ^k (x)-u_\ell ^k(y) \right) }{|x-y|^\frac{1}{2}}\psi (x)\chi _{|x-y| \ge \mathrm{dist\,}(D_1,\partial D)} \frac{\,\mathrm {d}x\,\mathrm {d}y}{|x-y|}\\&\quad = \int _{{\mathbb {R}}} \int _{{\mathbb {R}}} (\Omega _{D^c,\ell })_{jk}(x,y) (A_\ell (x))_{ij} \frac{\left( u_\ell ^k (x)-u_\ell ^k(y) \right) }{|x-y|^\frac{1}{2}}\psi (x)\chi _{|x-y| \ge \mathrm{dist\,}(D_1,\partial D)} \frac{\,\mathrm {d}x\,\mathrm {d}y}{|x-y|}. \end{aligned} \end{aligned}$$

We set

$$\begin{aligned} F_\ell (x,y) :=\chi _{|x-y| \ge \mathrm{dist\,}(D_1,\partial D)}\frac{\left( u_\ell (x)-u_\ell (y) \right) }{|x-y|^\frac{1}{2}} A_\ell (x) \psi (x) \end{aligned}$$

and

$$\begin{aligned} F(x,y):=\chi _{|x-y| \ge \mathrm{dist\,}(D_1,\partial D)}\frac{\left( u(x)-u(y) \right) }{|x-y|^\frac{1}{2}} A(x) \psi (x). \end{aligned}$$

We claim that we have the strong convergence

4.32 $$\begin{aligned} \lim _{\ell \rightarrow \infty } \Vert F_\ell - F\Vert _{L^{2}(\bigwedge \nolimits ^1_{od} {\mathbb {R}})} = 0. \end{aligned}$$

Indeed, we have

4.33 $$\begin{aligned} \begin{aligned}&\int _{{\mathbb {R}}}\int _{{\mathbb {R}}}|F_\ell (x,y)-F(x,y)|^2 \frac{\,\mathrm {d}x\,\mathrm {d}y}{|x-y|}\\&\quad \le \int _{{\mathbb {R}}}\int _{D_1}\left| d_\frac{1}{2} u_\ell (x,y) A_\ell (x) - d_\frac{1}{2} u(x,y)A(x) \right| ^2{|\psi (x)|^2}\chi _{|x-y| \ge \mathrm{dist\,}(D_1,\partial D)} \frac{\,\mathrm {d}x \,\mathrm {d}y}{|x-y|}\\&\quad \precsim \int _{{\mathbb {R}}}\int _{D_1} \left| d_\frac{1}{2} u_\ell (x,y) - d_\frac{1}{2} u(x,y) \right| ^2 \left( |A(x)|^2+|A_\ell (x)|^2 \right) {|\psi (x)|^2}\chi _{|x-y| \ge \mathrm{dist\,}(D_1,\partial D)} \frac{\,\mathrm {d}x \,\mathrm {d}y}{|x-y|}\\&\qquad + \int _{{\mathbb {R}}}\int _{D_1} \left| d_\frac{1}{2} u(x,y) \right| ^2|A_\ell (x) - A(x)|^2 |\psi (x)|^2 \chi _{|x-y| \ge \mathrm{dist\,}(D_1,\partial D)} \frac{\,\mathrm {d}x \,\mathrm {d}y}{|x-y|}. \end{aligned} \end{aligned}$$

For the first term of the right-hand side of (4.33), we take $R \gg 1$, such that in particular $\mathrm{supp\,}\psi \subset D_1 \subset \subset D \subset B(R)$ and estimate

4.34 $$\begin{aligned} \begin{aligned}&\int _{{\mathbb {R}}}\int _{D_1} \left| d_\frac{1}{2} u_\ell (x,y) - d_\frac{1}{2} u(x,y) \right| ^2 \left( |A(x)|^2 + |A_\ell (x)|^2 \right) {|\psi (x)|^2}\chi _{|x-y| \ge \mathrm{dist\,}(D_1,\partial D)} \frac{\,\mathrm {d}x \,\mathrm {d}y}{|x-y|}\\&\quad =\int _{{\mathbb {R}}\setminus B(R)}\int _{D_1} \left| d_\frac{1}{2} u_\ell (x,y) - d_\frac{1}{2} u(x,y) \right| ^2 \left( |A(x)|^2 \right. \\&\qquad \left. +\, |A_\ell (x)|^2\right) {|\psi (x)|^2}\chi _{|x-y| \ge \mathrm{dist\,}(D_1,\partial D)} \frac{\,\mathrm {d}x \,\mathrm {d}y}{|x-y|}\\&\qquad +\, \int _{B(R)}\int _{D_1} \left| d_\frac{1}{2} u_\ell (x,y) - d_\frac{1}{2} u(x,y) \right| ^2 \left( |A(x)|^2 \right. \\&\qquad \left. +\, |A_\ell (x)|^2\right) {|\psi (x)|^2}\chi _{|x-y| \ge \mathrm{dist\,}(D_1,\partial D)} \frac{\,\mathrm {d}x \,\mathrm {d}y}{|x-y|}. \end{aligned} \end{aligned}$$

Now, for the second term of the right-hand side of (4.34) we have

$$\begin{aligned} \begin{aligned}&\int _{B(R)}\int _{D_1} \left| d_\frac{1}{2} u_\ell (x,y) - d_\frac{1}{2} u(x,y) \right| ^2 \left( |A(x)|^2 \right. \\&\qquad \left. +\, |A_\ell (x)|^2\right) {|\psi (x)|^2}\chi _{|x-y| \ge \mathrm{dist\,}(D_1,\partial D)} \frac{\,\mathrm {d}x \,\mathrm {d}y}{|x-y|}\\&\quad \precsim \left( \Vert A\Vert _{L^\infty (D_1)}^2+\Vert A_\ell \Vert _{L^\infty (D_1)}^2 \right) \Vert \psi \Vert ^2_{L^\infty } \int _{{B(R)}}\\&\qquad \int _{D_1} \frac{|u_\ell (x) - u(x)|^2 + |u_\ell (y)-u(y)|^2}{|x-y|^2} \,\mathrm {d}x \,\mathrm {d}y\\&\quad \precsim \left( \Vert A\Vert _{L^\infty (D_1)}^2+\Vert A_\ell \Vert _{L^\infty (D_1)}^2 \right) \Vert \psi \Vert ^2_{L^\infty }\mathrm{dist\,}^{-2}(D_1,\partial D)\bigg (\int _{{B(R)}}\int _{D_1} |u_\ell (x) - u(x)|^2 \,\mathrm {d}x \,\mathrm {d}y\\&\qquad + \int _{{B(R)}}\int _{D_1}|u_\ell (y)-u(y)|^2\,\mathrm {d}x \,\mathrm {d}y\bigg )\\&\quad \le C(D_1,D,R) \left( \Vert A\Vert _{L^\infty (D_1)}^2+\Vert A_\ell \Vert _{L^\infty (D_1)}^2 \right) \Vert u_\ell - u\Vert ^2_{L^2(B(R))}. \end{aligned} \end{aligned}$$

Thus, by the strong convergence on compact sets of $u_\ell$ in $L^2$ we obtain

4.35 $$\begin{aligned}&\lim _{\ell \rightarrow \infty } \int _{B(R)}\int _{D_1} \left| d_\frac{1}{2} u_\ell (x,y) - d_\frac{1}{2} u(x,y) \right| ^2 \nonumber \\&\quad \left( |A(x)|^2 + |A_\ell (x)|^2 \right) {|\psi (x)|^2}\chi _{|x-y| \ge \mathrm{dist\,}(D_1,\partial D)} \frac{\,\mathrm {d}x \,\mathrm {d}y}{|x-y|} =0. \end{aligned}$$

Now we estimate the first term of the right-hand side of (4.34). We observe that for all large *R*, whenever $x \in \mathrm{supp\,}\psi$ and $y \not \in B(R)$, we have $|x-y| \succsim 1+|y|$. Therefore,

$$\begin{aligned} \begin{aligned}&\int _{{\mathbb {R}}\setminus B(R)}\int _{D_1} \left| d_\frac{1}{2} u_\ell (x,y) - d_\frac{1}{2} u(x,y) \right| ^2 \left( |A(x)|^2 \right. \\&\qquad \left. +\, |A_\ell (x)|^2\right) {|\psi (x)|^2}\chi _{|x-y| \ge \mathrm{dist\,}(D_1,\partial D)} \frac{\,\mathrm {d}x \,\mathrm {d}y}{|x-y|}\\&\quad \precsim \left( \Vert A\Vert _{L^\infty (D_1)}^2+\Vert A_\ell \Vert _{L^\infty (D_1)}^2 \right) \Vert \psi \Vert ^2_{L^\infty } \int _{{\mathbb {R}}\setminus B(R)}\\&\qquad \int _{D_1} \frac{|u_\ell (x) - u(x)|^2 + |u_\ell (y) - u(y)|^2}{1+|y|^2} \,\mathrm {d}x\,\mathrm {d}y\\&\quad \precsim \left( \Vert A\Vert _{L^\infty (D_1)}^2+\Vert A_\ell \Vert _{L^\infty (D_1)}^2 \right) \Vert \psi \Vert ^2_{L^\infty } \Vert u_\ell - u\Vert _{L^2(D_1)}^2 \int _{{\mathbb {R}}\setminus B(R)} \frac{1}{1+|y|^2} \,\mathrm {d}y\\&\qquad + \left( \Vert A\Vert _{L^\infty (D_1)}^2+\Vert A_\ell \Vert _{L^\infty (D_1)}^2 \right) \Vert \psi \Vert ^2_{L^\infty } \Vert u_\ell \\&\qquad - u\Vert _{L^2 + L^\infty ({\mathbb {R}})}^{{2}}\max \left\{ \int _{{\mathbb {R}}\setminus B(R)}\frac{1}{1+|y|^2}\,\mathrm {d}y , \frac{1}{1+R^2}\right\} \\&\quad \precsim R^{{-1}} \left( \Vert A\Vert _{L^\infty (D_1)}^2+\Vert A_\ell \Vert _{L^\infty (D_1)}^2 \right) \Vert \psi \Vert ^2_{L^\infty } \Vert u_\ell - u\Vert ^{{ 2}}_{L^2 + L^\infty ({\mathbb {R}})}. \end{aligned} \end{aligned}$$

Thus,

4.36 $$\begin{aligned}&\lim _{R\rightarrow \infty } \sup _{\ell } \int _{{\mathbb {R}}\setminus B(R)}\int _{D_1} \left| d_\frac{1}{2} u_\ell (x,y) - d_\frac{1}{2} u(x,y) \right| ^2 \nonumber \\&\quad \left( |A(x)|^2 + |A_\ell (x)|^2 \right) {|\psi (x)|^2}\chi _{|x-y| \ge \mathrm{dist\,}(D_1,\partial D)} \frac{\,\mathrm {d}x \,\mathrm {d}y}{|x-y|} =0. \end{aligned}$$

Combining (4.34) with (4.35) and (4.36), we obtain the convergence of the first term of the right-hand side of (4.33)

4.37 $$\begin{aligned}&\lim _{\ell \rightarrow \infty }\int _{{\mathbb {R}}}\int _{D_1} \left| d_\frac{1}{2} u_\ell (x,y) - d_\frac{1}{2} u(x,y) \right| ^2 \nonumber \\&\quad \left( |A(x)|^2 + {|A_\ell (x)|^2} \right) {|\psi (x)|^2}\chi _{|x-y| \ge \mathrm{dist\,}(D_1,\partial D)} \frac{\,\mathrm {d}x \,\mathrm {d}y}{|x-y|} =0. \end{aligned}$$

*As for the second term of the right-hand side of* (4.33), we observe that since $A_\ell \rightarrow A$ pointwise almost everywhere, we have

$$\begin{aligned} \lim _{\ell \rightarrow \infty }\left| d_\frac{1}{2} u(x,y) \right| ^2|A_\ell (x) - A(x)|^2 |\psi (x)|^2 \frac{\chi _{|x-y| \ge \mathrm{dist\,}(D_1,\partial D)} }{|x-y|} = 0 \quad \text { pointwise a.e. in } D_1 \times {\mathbb {R}}. \end{aligned}$$

Moreover, we have

$$\begin{aligned} \begin{aligned}&\left| d_\frac{1}{2} u(x,y) \right| ^2|A_\ell (x) - A(x)|^2 |\psi (x)|^2 \chi _{|x-y| \ge \mathrm{dist\,}(D_1,\partial D)} \frac{1}{|x-y|}\\&\quad \precsim \left( \sup _{\ell } \Vert A_\ell \Vert ^{{2}}_{L^\infty }+ \Vert A\Vert ^{{2}}_{L^\infty } \right) \left| d_\frac{1}{2} u(x,y) \right| ^2 |\psi (x)|^2 \chi _{|x-y| \ge \mathrm{dist\,}(D_1,\partial D)} \frac{1}{|x-y|} \end{aligned} \end{aligned}$$

and the right-hand side is independent of $\ell$ and integrable. Thus, by dominated convergence theorem we have

4.38 $$\begin{aligned} \lim _{\ell \rightarrow \infty } \int _{{\mathbb {R}}}\int _{D_1} \left| d_\frac{1}{2} u(x,y) \right| ^2|A_\ell (x) - A(x)|^2 |\psi (x)|^2 \chi _{|x-y| \ge \mathrm{dist\,}(D_1,\partial D)} \frac{\,\mathrm {d}x \,\mathrm {d}y}{|x-y|} = 0. \end{aligned}$$

Now, plugging (4.38) and (4.37) into (4.33) we establish (4.32).

Thus, (4.32) and a combination of the weak convergence of $\Omega _{\ell ,D^c}$ and the strong convergence of $F_\ell$ implies

$$\begin{aligned} \lim _{\ell \rightarrow \infty } \int _{\mathbb {R}}\Omega _{\ell , D^c}(x,y) F_\ell (x,y) \frac{\,\mathrm {d}x\,\mathrm {d}y}{|x-y|} = \int _{\mathbb {R}}\Omega _{D^c}(x,y) F(x,y) \frac{\,\mathrm {d}x\,\mathrm {d}y}{|x-y|}. \end{aligned}$$

This establishes (4.30).

*Step 5.* We claim that

4.39 $$\begin{aligned} \lim _{\ell \rightarrow \infty } f_\ell [A_\ell \psi ] = f[A\psi ]. \end{aligned}$$

Indeed, this holds because $A_\ell \psi$ is uniformly bounded in ${{\dot{W}}}^{\frac{1}{2},2}$, $A_\ell \psi$ converges weakly to $A\psi$ in ${\dot{W}}^{\frac{1}{2},2}$, and by assumption $f_\ell \rightarrow f$ in ${{W}}^{-\frac{1}{2},2}$.

*Step 6.* Passing to the limit.

Passing with $\ell \rightarrow \infty$ in (4.5), using (4.7), (4.15), (4.30), and (4.39), we obtain

4.40 $$\begin{aligned} \int _{{\mathbb {R}}} A d_\frac{1}{2} u \cdot d_\frac{1}{2} \psi = \int _{{\mathbb {R}}} \left( \Omega _{D}^{A} \right) ^*\cdot u d_{\frac{1}{2}}\psi +\int _{{\mathbb {R}}} A \Omega _{D^c}\cdot d_{\frac{1}{2}} u \, \psi + f[A \psi ]. \end{aligned}$$

By (4.15) we know that $\left( \Omega _{D}^{A} \right) ^*$ is $\frac{1}{2}$-divergence free and thus by Lemma 2.1 we have

$$\begin{aligned} \int _{{\mathbb {R}}} \left( \Omega _{D}^{A} \right) ^*\cdot u d_{\frac{1}{2}}\psi = \int _{{\mathbb {R}}} \Omega _{D}^{A}\cdot d_\frac{1}{2} u\psi , \end{aligned}$$

which combined with (4.40) and formulas $\Omega _D^A = A\Omega _D - d_\frac{1}{2}A$ and $\Omega _{D^c}=\Omega -\Omega _D$ gives

4.41 $$\begin{aligned} \int _{{\mathbb {R}}} A d_\frac{1}{2} u \cdot d_\frac{1}{2} \psi = \int _{{\mathbb {R}}} \Omega ^{A}\cdot d_{\frac{1}{2}}u\psi + f[A \psi ]. \end{aligned}$$

This holds for any $\psi \in C_c^\infty (D_1)$. By density we can invoke Lemma 4.1, which leads to the claim. $\square$

### Corollary 4.3

Let $u_\ell$, $\Omega _\ell$, and $f_\ell$ be as in Theorem 1.3. Let $D\subset {\mathbb {R}}$. Then there exists a locally finite $\Sigma \subset D$ such that

$$\begin{aligned} (-\Delta )^{\frac{1}{2}} u = \Omega \cdot d_\frac{1}{2} u +f\quad \text {in } D \setminus \Sigma . \end{aligned}$$

### Proof

We follow in spirit the covering argument of Sacks–Uhlenbeck [30, Proposition 4.3 & Theorem 4.4].

By assumptions there is a number $\Lambda >0$ such that $\sup _{\ell \in {{\mathbb {N}}}} \Vert \Omega _\ell \Vert _{L^2(\bigwedge \nolimits _{od}^1{\mathbb {R}})}<\Lambda$.

Let $\alpha \in {{\mathbb {N}}}$ and let $\mathcal {B}_\alpha :=\{B(x_{i,\alpha },2^{-\alpha }):x_{i,\alpha }\in D\}$ be a family of balls such that $D\subset \bigcup \mathcal {B}_\alpha$ and each point $x\in D$ is covered at most $\lambda$ times, and such that for a smaller radius we still have $D \subset \bigcup _{i} B(x_{i,\alpha }, 2^{-\alpha -1})$. Then

$$\begin{aligned} \sum _{i} \int _{B(x_{i,\alpha },2^{-\alpha })}\int _{{\mathbb {R}}} |\Omega _\ell (x,y)|^2 \frac{\,\mathrm {d}x \,\mathrm {d}y}{|x-y|} < \Lambda \lambda . \end{aligned}$$

Now, let $\sigma >0$ be the number from Theorem 1.1, then there exists at most $\frac{\Lambda \lambda }{\sigma }$ balls in $\mathcal {B}_\alpha$ on which

$$\begin{aligned} \int _{B(x_{i,\alpha },2^{-\alpha })}\int _{{\mathbb {R}}} |\Omega _\ell (x,y)|^2 \frac{\,\mathrm {d}x \,\mathrm {d}y}{|x-y|} > \sigma . \end{aligned}$$

Thus, by Proposition 4.2, we obtain that except for $K<\frac{\Lambda \lambda }{\sigma } +1$ balls from $\mathcal {B}_\alpha$ we have

4.42 $$\begin{aligned} \int _{\mathbb {R}}d_\frac{1}{2} u \cdot d_\frac{1}{2} \varphi _i = \int _{\mathbb {R}}\Omega \cdot d_\frac{1}{2} u \varphi _i + f[\varphi _i]\quad \text { for all } \varphi _i\in C_c^\infty (B(x_{i,\alpha },2^{-\alpha -1})). \end{aligned}$$

Let us denote those balls by $B(y_{i,\alpha }, 2^{-\alpha })$ for $i=1,\dots , K$. Then by (4.42) we get

4.43 $$\begin{aligned} \int _{\mathbb {R}}d_\frac{1}{2} u \cdot d_\frac{1}{2} \psi = \int _{\mathbb {R}}\Omega \cdot d_\frac{1}{2} u \psi + f[\psi ], \quad \text { for all }\psi \in C_c^\infty \left(D\setminus \bigcup _{i\le K} {\overline{B}}(y_{i,\alpha },2^{-\alpha -1})\right). \end{aligned}$$

Since $\bigcup _{\alpha \in {{\mathbb {N}}}}\left( D\setminus \bigcup _{i=1}^K {\overline{B}}(y_{i,\alpha },2^{-\alpha -1}) \right) = D\setminus \{x_1,\ldots ,x_K\}$, (4.43) holds for any $\psi \in C_c^\infty (D\setminus \Sigma )$, where $\Sigma :=\{x_1,\ldots ,x_K\}$. This gives the claim. $\square$

In order to conclude we will need a removability of singularities lemma, compare with [18, Proposition 4.7].

### Lemma 4.4

Let $u \in {\dot{W}}^{\frac{1}{2},2}({\mathbb {R}},{\mathbb {R}}^N)$, $f \in L^1({\mathbb {R}},{\mathbb {R}}^N)$, and $g \in W^{-\frac{1}{2},2}({\mathbb {R}})$. Assume that for some locally finite set $\Sigma \subset D$ we have

$$\begin{aligned} (-\Delta )^\frac{1}{2} u = f +g \quad \text {in }D \setminus \Sigma . \end{aligned}$$

Then

$$\begin{aligned} (-\Delta )^\frac{1}{2} u = f +g\quad \text {in }D. \end{aligned}$$

### Proof

For simplicity of presentation let us assume that $\Sigma =\{x_0\}$. By definition we have for any $\varphi \in C^\infty _c(D\setminus \{x_0\})$

$$\begin{aligned} \int _D \int _D \frac{(u(x) - u(y))(\varphi (x) - \varphi (y))}{|x-y|^{2}} \,\mathrm {d}x \,\mathrm {d}y = \int _D f(x) \varphi (x) \,\mathrm {d}x+g[\varphi ]. \end{aligned}$$

Let $\{\zeta _\ell \}_{\ell \in {{\mathbb {N}}}} \subset C_c^\infty (D,[0,1])$ be the sequence from Lemma D.1, i.e., such that for all $\ell \in {{\mathbb {N}}}$ we have

4.44 $$\begin{aligned} \zeta _\ell \equiv 1 \text { on } B_{\rho _\ell }(x_0), \quad \zeta _\ell \equiv 0 \text { outside } B_{R_\ell }(x_0), \quad \text { and }\lim _{\ell \rightarrow \infty }[\zeta _\ell ]_{W^{\frac{1}{2},2}(D)}=0 \end{aligned}$$

for a $$0<\rho _\ell <R_\ell \rightarrow 0$$ as $$\ell \rightarrow \infty $$.

Now let $\psi \in C^\infty _c(D)$ and then $\psi _\ell :=\psi (1-\zeta _\ell )\in C_c^\infty (\Sigma \setminus \{x_0\})$ is an admissible test function and we have

4.45 $$\begin{aligned} \int _D \int _D \frac{(u(x) - u(y))(\psi (x)-\psi (y))}{|x-y|^{2}} \,\mathrm {d}x \,\mathrm {d}y - \mathcal {I}_\ell = \int _D f(x) \psi (x)\,\mathrm {d}x + g[\psi ] - \mathcal {II}_\ell - \mathcal {III}_{\ell }. \end{aligned}$$

We have

4.46 $$\begin{aligned} \begin{aligned} \mathcal {I}_\ell&:=\int _D \int _D \frac{(u(x) - u(y))(\psi (x)\zeta _\ell (x) - \psi (y)\zeta _\ell (y))}{|x-y|^{2}} \,\mathrm {d}x \,\mathrm {d}y\\&= \int _D \int _D \frac{(u(x) - u(y))\psi (x)(\zeta _\ell (x) - \zeta _\ell (y))}{|x-y|^{2}} \,\mathrm {d}x \,\mathrm {d}y\\&\quad + \int _D \int _D \frac{(u(x) - u(y))(\psi (x)-\psi (y))\zeta _\ell (y)}{|x-y|^{2}} \,\mathrm {d}x \,\mathrm {d}y\\&\le \Vert \psi \Vert _{L^\infty (D)} [u]_{W^{\frac{1}{2},2}(D)}[\zeta _\ell ]_{W^{\frac{1}{2},2}(D)}+ \int _{B_{R_\ell }}\int _{D}\frac{{|}u(x) - u(y){||}\psi (x)-\psi (y){|}}{|x-y|^{2}} \,\mathrm {d}x \,\mathrm {d}y{.} \end{aligned} \end{aligned}$$

Thus, by (4.44) and by the absolute continuity of the integral we have $\lim _{\ell \rightarrow \infty } \mathcal {I}_\ell =0$.

Secondly,

4.47 $$\begin{aligned} \mathcal {II}_\ell :=\int _D f(x) \psi (x)\zeta _\ell (x) \,\mathrm {d}x\le \Vert \psi \Vert _{L^\infty }\int _{B_{R_\ell }} |f(x)| \,\mathrm {d}x \xrightarrow {\ell \rightarrow \infty } 0, \end{aligned}$$

by the absolute continuity of the integral.

Thus, passing with $\ell \rightarrow \infty$ in (4.45) we get for any $\psi \in C_c^\infty (D)$

$$\begin{aligned} \int _D \int _D \frac{(u(x) - u(y))(\psi (x)-\psi (y))}{|x-y|^{2}} \,\mathrm {d}x \,\mathrm {d}y = \int _D f(x) \psi (x)\,\mathrm {d}x. \end{aligned}$$

Lastly,

$$\begin{aligned} \mathcal {III}_\ell :=g[\psi \,\zeta _\ell ] \xrightarrow {\ell \rightarrow \infty } 0, \end{aligned}$$

because, by (4.44), we have $[\psi \,\zeta _\ell ]_{W^{\frac{1}{2},2}} \xrightarrow {\ell \rightarrow \infty } 0$.

This finishes the proof. $\square$

### Proof of Theorem 1.3

Combining Corollary 4.3 and Lemma 4.4, we obtain the claim. $\square$

## Appendix A: Nonlocal Hodge decomposition

### Lemma A.1

Let $p>1$, $s\in (0,1)$, $G \in L^p(\bigwedge \nolimits _{od}^1 {\mathbb {R}}^n)$ then there exists a decomposition^1^

$$\begin{aligned} G = d_{s} a + B, \end{aligned}$$

where $a \in {\dot{W}}^{s,p}({\mathbb {R}}^n)$ and $B \in L^p(\bigwedge \nolimits _{od} ^1{\mathbb {R}}^n)$ with ${\text {div}}_{s} B = 0$. Moreover,

A.1 $$\begin{aligned} \Vert B\Vert _{L^p(\bigwedge \nolimits _{od}^1 {\mathbb {R}}^n)} + [a]_{W^{s,p}({\mathbb {R}}^n)} \precsim \Vert G\Vert _{L^p(\bigwedge \nolimits _{od}^1{\mathbb {R}}^n)}. \end{aligned}$$

### Proof

Since $G\in L^p(\bigwedge \nolimits _{od}^1 {\mathbb {R}}^n)$ we have ${\text {div}}_s G \in \left( W^{s,p'}({\mathbb {R}}^n) \right) ^*$, namely

$$\begin{aligned} {\text {div}}_s G[\varphi ] \precsim \Vert G\Vert _{L^p(\bigwedge \nolimits _{od}^1{\mathbb {R}}^n)}\, [\varphi ]_{W^{s,p'}({\mathbb {R}}^n)}. \end{aligned}$$

Recall that for $0<s<1$ and $1\le p <\infty$ we have ${\dot{W}}^{s,p}({\mathbb {R}}^n) = {\dot{F}}^s_{p,p}({\mathbb {R}}^n)$ [34, 2.3.5]. Moreover, ${\text {div}}_s G \in {\dot{F}}^{-s}_{p,p}$, since $(-\Delta )^{-s}: {\dot{F}}^{{s}}_{p,p}({\mathbb {R}}^n) \rightarrow {\dot{F}}^{{-s}}_{p,p}({\mathbb {R}}^n)$ is an isomorphism [29, §2.6.2, Proposition 2, p.95]. In particular, there is a unique solution $a \in {\dot{F}}^s_{p,p}({\mathbb {R}}^n)$ to the distributional equation

$$\begin{aligned} (-\Delta )^s a = {\text {div}}_{s} G. \end{aligned}$$

with

$$\begin{aligned}{}[a]_{{\dot{F}}^s_{p,p}({\mathbb {R}}^n)} \precsim [{\text {div}}_s G]_{F^{-s}_{p',p'}({\mathbb {R}}^n)} \precsim \Vert G\Vert _{L^p(\bigwedge \nolimits _{od}^1 {\mathbb {R}}^n)}. \end{aligned}$$

We have found $a \in {\dot{F}}^{s}_{p,p}({\mathbb {R}}^n) = {\dot{W}}^{s,p}({\mathbb {R}}^n)$, and we have

$$\begin{aligned} \int _{{\mathbb {R}}^n} d_s a \cdot d_s \varphi = \int _{{\mathbb {R}}^n} F \varphi \quad \forall \varphi \in C_c^\infty ({\mathbb {R}}^n). \end{aligned}$$

The uniqueness of *a* up to a normalization assumption would follow by considering a difference of two solutions and an application of nonlocal Liouville theorem [11, Theorem 1.1].

Now define $B:=G - d_s a$. We have

$$\begin{aligned} {\text {div}}_s B = {\text {div}}_s G - {\text {div}}_s (d_s a) = {\text {div}}_s G - (-\Delta )^s a =0, \end{aligned}$$

which finishes the proof. $\square$

## Appendix B: Localization

The next proposition follows from a relatively straightforward localization results, see, e.g., [19].

### Proposition B.1

Assume $D_1 \subset \subset D_2 \subset \subset D' \subseteq D \subseteq {\mathbb {R}}$ open intervals and let $u \in L^1({\mathbb {R}},{\mathbb {R}}^N) + L^\infty ({\mathbb {R}},{\mathbb {R}}^N)\cap {\dot{W}}^{{\frac{1}{2}},2}(D,{\mathbb {R}}^N)$ be a solution to

$$\begin{aligned} (-\Delta )^{\frac{1}{2}}_{D} u = \Omega \cdot _{D} d_{\frac{1}{2}} u + f \quad \text {in } D'. \end{aligned}$$

That is, assume

B.1 $$\begin{aligned} \begin{aligned}&\int _{D} \int _{D} \frac{(u(x)-u(y)) (\varphi (x)-\varphi (y))}{|x-y|^{2}}{\,\mathrm {d}x\,\mathrm {d}y}\\&\quad = \int _{D} \int _{D} \Omega (x,y) d_{\frac{1}{2}} u(x,y) \varphi (x)\, \frac{\,\mathrm {d}x\,\mathrm {d}y}{|x-y|} + \int _{D} f \varphi , \quad \forall \varphi \in C_c^\infty (D'). \end{aligned} \end{aligned}$$

Let $\eta \in C_c^\infty (D_1)$ and set $v :=\eta u$ and ${\tilde{\Omega }}_{ij}(x,y) = \chi _{D_2}(x)\chi _{D_2}(y)\Omega _{ij}(x,y)$. Then

$$\begin{aligned} (-\Delta )^{\frac{1}{2}} v = {\tilde{\Omega }} \cdot d_{\frac{1}{2}} v + \eta f + \mathcal {G}(u,\cdot ) \quad \text {in }{\mathbb {R}}, \end{aligned}$$

where $\mathcal {G}$ is a bilinear form with the following estimates for any $s \in (0,\frac{1}{2})$ and $\varepsilon > 0$

$$\begin{aligned} \begin{aligned} |\mathcal {G}(u,{\varphi })| \le&\,C(\eta ,s,\varepsilon ,D_1,D_2)\, \left( 1+\Vert \Omega \Vert _{L^2(\bigwedge \nolimits ^1_{od} D)} \right) \\&\cdot \left( \Vert u\Vert _{L^2(D)+L^\infty (D)} + [u]_{W^{s,2}(D_2)} \right) \\&\cdot \left( \Vert \varphi \Vert _{L^2(D)+L^\infty (D)} + \Vert \varphi \Vert _{L^{\frac{1}{s}}(D_2)} + \Vert \varphi \Vert _{L^1 + L^\infty ({\mathbb {R}})} + [\varphi ]_{W^{\varepsilon ,\frac{2}{2s+1}}(D_2)} \right) . \end{aligned} \end{aligned}$$

In particular we have

$$\begin{aligned} \Vert {\tilde{\Omega }}\Vert _{L^2(\bigwedge \nolimits ^1_{od} {\mathbb {R}})} \le \Vert \Omega \Vert _{L^2(\bigwedge \nolimits ^1_{od} D_2)}. \end{aligned}$$

### Proof

Let $\varphi \in C_c^\infty ({\mathbb {R}})$. We have

$$\begin{aligned} \begin{aligned}&\left( \eta (x) u(x) - \eta (y) u(y) \right) \left( \varphi (x) -\varphi (y) \right) \\&\quad =(u(x)-u(y)) (\eta (x) \varphi (x) - \eta (y) \varphi (y)) +(\eta (x)-\eta (y)) \left( u(y) \varphi (x) - u(x) \varphi (y) \right) . \end{aligned} \end{aligned}$$

Since $\eta \varphi \in C_c^\infty (D')$ it is an admissible test function and we have from the Eq. (B.1)

$$\begin{aligned} \begin{aligned}&\int _{D} \int _{D} \frac{(v(x)-v(y)) (\varphi (x)-\varphi (y))}{|x-y|^{2}}\,\mathrm {d}x\,\mathrm {d}y\\&\quad = \int _{D} \int _{D} \Omega (x,y) d_{\frac{1}{2}} u(x,y) \eta (x)\varphi (x)\, \frac{\,\mathrm {d}x\,\mathrm {d}y}{|x-y|} + \int _{{\mathbb {R}}} f \eta \varphi +\mathcal {G}_1(u,\varphi ). \end{aligned} \end{aligned}$$

Here,

$$\begin{aligned} \mathcal {G}_1(u,\varphi ) = \int _{D} \int _{D} \frac{(\eta (x)-\eta (y)) \left( u(y) \varphi (x) - u(x) \varphi (y) \right) }{|x-y|^{2}}{\,\mathrm {d}x\,\mathrm {d}y}. \end{aligned}$$

Moreover, we have

$$\begin{aligned} \begin{aligned}&\int _{{\mathbb {R}}} \int _{{\mathbb {R}}} \frac{(v(x)-v(y)) (\varphi (x)-\varphi (y))}{|x-y|^{2}}{\,\mathrm {d}x\,\mathrm {d}y}\\&\quad = \int _{D} \int _{D} \frac{(v(x)-v(y)) (\varphi (x)-\varphi (y))}{|x-y|^{2}}{\,\mathrm {d}x\,\mathrm {d}y} + \mathcal {G}_2(u,\varphi ), \end{aligned} \end{aligned}$$

where, because $$\mathrm{supp\,}v \subset D_1$$,

$$\begin{aligned} \begin{aligned} \mathcal {G}_2(u,\varphi ) = 2\int _{D_1} v(x) \int _{{\mathbb {R}}\setminus D} \frac{ (\varphi (x)-\varphi (y))}{|x-y|^{2}}{{ \,\mathrm {d}y\,\mathrm {d}x}}. \end{aligned} \end{aligned}$$

That is we have

$$\begin{aligned} \begin{aligned}&\int _{{\mathbb {R}}} \int _{{\mathbb {R}}} \frac{(v(x)-v(y)) (\varphi (x)-\varphi (y))}{|x-y|^{2}}{\,\mathrm {d}x\,\mathrm {d}y}\\&\quad = \int _{D} \int _{D} \Omega (x,y) d_{\frac{1}{2}} u(x,y) \eta (x)\varphi (x)\, \frac{\,\mathrm {d}x\,\mathrm {d}y}{|x-y|} + \int _{{\mathbb {R}}} f \eta \varphi +\mathcal {G}_1(u,\varphi )+\mathcal {G}_2(u,\varphi ). \end{aligned} \end{aligned}$$

Furthermore, since

$$\begin{aligned} d_{\frac{1}{2}}u(x,y) \eta (x) = d_{\frac{1}{2}} (\eta u)(x,y) - u(y) d_{\frac{1}{2}}\eta (x,y) \end{aligned}$$

and $\mathrm{supp\,}v\subset D_1$, we have

$$\begin{aligned} \begin{aligned}&\int _{D} \int _{D} \Omega (x,y) d_{\frac{1}{2}} u(x,y) \eta (x)\varphi (x)\, \frac{\,\mathrm {d}x\,\mathrm {d}y}{|x-y|}\\&\quad =\int _{D} \int _{D} \Omega (x,y) d_{\frac{1}{2}} v(x,y)\, \varphi (x)\, \frac{\,\mathrm {d}x\,\mathrm {d}y}{|x-y|} -\int _{D} \int _{D} \Omega (x,y) u(y) d_{\frac{1}{2}}\eta (x,y)\varphi (x)\, \frac{\,\mathrm {d}x\,\mathrm {d}y}{|x-y|}\\&\quad =\int _{{\mathbb {R}}} \int _{{\mathbb {R}}} \chi _{D_2}(x) \chi _{D_2}(y)\Omega (x,y) d_{\frac{1}{2}} v(x,y)\, \varphi (x)\, \frac{\,\mathrm {d}x\,\mathrm {d}y}{|x-y|}\\&\qquad +\int _{D \setminus D_2} \int _{D_2} \Omega (x,y) d_{\frac{1}{2}} v(x,y)\, \varphi (x)\, \frac{\,\mathrm {d}x\,\mathrm {d}y}{|x-y|}\\&\qquad +\int _{D_2} \int _{D \setminus D_2} \Omega (x,y) d_{\frac{1}{2}} v(x,y)\, \varphi (x)\, \frac{\,\mathrm {d}x\,\mathrm {d}y}{|x-y|}\\&\qquad -\int _{D} \int _{D} \Omega (x,y) u(y) d_{\frac{1}{2}}\eta (x,y)\varphi (x)\, \frac{\,\mathrm {d}x\,\mathrm {d}y}{|x-y|}. \end{aligned} \end{aligned}$$

So if we set

$$\begin{aligned} \mathcal {G}_3(u,\varphi ):= & {} \int _{D \setminus D_2} \int _{D_2} \Omega (x,y) d_{\frac{1}{2}} v(x,y)\, \varphi (x)\, \frac{\,\mathrm {d}x\,\mathrm {d}y}{|x-y|} \\&+ \int _{D_2} \int _{D \setminus D_2} \Omega (x,y) d_{\frac{1}{2}} v(x,y)\, \varphi (x)\, \frac{\,\mathrm {d}x\,\mathrm {d}y}{|x-y|} \end{aligned}$$

and

$$\begin{aligned} \mathcal {G}_4(u,\varphi ) :=-\int _{D} \int _{D} \Omega (x,y) u(y) d_{\frac{1}{2}}\eta (x,y)\varphi (x)\, \frac{\,\mathrm {d}x\,\mathrm {d}y}{|x-y|}, \end{aligned}$$

then we have shown for any $\varphi \in C_c^\infty ({\mathbb {R}})$,

$$\begin{aligned} \begin{aligned} \int _{{\mathbb {R}}} \int _{{\mathbb {R}}} \frac{(v(x)-v(y)) (\varphi (x)-\varphi (y))}{|x-y|^{2}}{\,\mathrm {d}x\,\mathrm {d}y} = \int _{{\mathbb {R}}} {\tilde{\Omega }}\cdot d_{\frac{1}{2}} v \, \varphi + \int _{{\mathbb {R}}} f \eta \varphi +\sum _{i=1}^4\mathcal {G}_i(u,\varphi ). \end{aligned} \end{aligned}$$

It remains to estimate each $\mathcal {G}_i(u,\varphi )$.

${{\textit{Estimate of }\mathcal {G}_1:}}$ By the support of $\eta$ we have

B.2 $$\begin{aligned} \begin{aligned} \mathcal {G}_1(u,\varphi )&= \int _{D_2} \int _{D_2} \frac{(\eta (x)-\eta (y)) \left( u(y) \varphi (x) - u(x) \varphi (y) \right) }{|x-y|^{2}}{\,\mathrm {d}x\,\mathrm {d}y}\\&\quad + 2 \int _{D_1}\int _{D\setminus D_2}\frac{(\eta (x)-\eta (y)) \left( u(y) \varphi (x) - u(x) \varphi (y) \right) }{|x-y|^{2}}{\,\mathrm {d}x\,\mathrm {d}y}. \end{aligned} \end{aligned}$$

As for the first term, we have

B.3 $$\begin{aligned} \begin{aligned}&\int _{D_2} \int _{D_2} \frac{(\eta (x)-\eta (y)) \left( u(y) \varphi (x) - u(x) \varphi (y) \right) }{|x-y|^{2}}{\,\mathrm {d}x\,\mathrm {d}y}\\&\quad \le \Vert \eta \Vert _{\mathrm{Lip\,}} \int _{D_2} \int _{D_2} \frac{|u(y) \varphi (x)-u(x)\varphi (y)|}{|x-y|}{\,\mathrm {d}x\,\mathrm {d}y}\\&\quad \precsim \Vert \eta \Vert _{\mathrm{Lip\,}} \left( \int _{D_2} |u(y)|\int _{D_2} \frac{|\varphi (x)-\varphi (y)|}{|x-y|}{\,\mathrm {d}x\,\mathrm {d}y}+ \int _{D_2} |\varphi (y)|\int _{D_2} \frac{|u(x)-u(y)|}{|x-y|}{\,\mathrm {d}x\,\mathrm {d}y} \right) \\&\quad \precsim \Vert \eta \Vert _{\mathrm{Lip\,}} \int _{D_2} |u(y)-(u)_{D_2}|\int _{D_2} \frac{|\varphi (x)-\varphi (y)|}{|x-y|}{\,\mathrm {d}x\,\mathrm {d}y}\\&\qquad +\Vert \eta \Vert _{\mathrm{Lip\,}}\Vert u\Vert _{L^1(D_2)} \int _{D_2} \int _{D_2} \frac{|\varphi (x)-\varphi (y)|}{|x-y|}{\,\mathrm {d}x\,\mathrm {d}y}\\&\qquad +\Vert \eta \Vert _{\mathrm{Lip\,}} \int _{D_2} |\varphi (y)|\int _{D_2} \frac{|u(x)-u(y)|}{|x-y|}{\,\mathrm {d}x\,\mathrm {d}y}. \end{aligned} \end{aligned}$$

We observe that for any $p \in (1,\infty )$ and any $\varepsilon > 0$ we have

$$\begin{aligned} \begin{aligned} \int _{D_2}\left( \int _{D_2} \frac{|\varphi (x)-\varphi (y)|}{|x-y|} \,\mathrm {d}x \right) ^p \,\mathrm {d}y&=\int _{D_2}\left( \int _{D_2} \frac{|\varphi (x)-\varphi (y)|}{|x-y|^\varepsilon } |x-y|^{\varepsilon }\frac{\,\mathrm {d}x}{|x-y|} \right) ^p \,\mathrm {d}y\\&\quad \precsim [\varphi ]_{W^{\varepsilon ,p}(D_2)}\,{\sup _{y\in D_2}} {\left( \int _{D_2} |x-y|^{\varepsilon p'} \frac{\,\mathrm {d}x}{|x-y|} \right) ^{\frac{p}{p'}} }\\&\quad \precsim C(D_2)[\varphi ]_{W^{\varepsilon ,p}(D_2)}. \end{aligned} \end{aligned}$$

Thus, for any $\varepsilon >0$ and any $s\in (0,\frac{1}{2})$ we have

B.4 $$\begin{aligned} \begin{aligned}&\int _{D_2} |u(y)-(u)_{D_2}|\int _{D_2} \frac{|\varphi (x)-\varphi (y)|}{|x-y|}{\,\mathrm {d}x\,\mathrm {d}y} + \Vert u\Vert _{L^1(D_2)} \int _{D_2} \int _{D_2} \frac{|\varphi (x)-\varphi (y)|}{|x-y|}{\,\mathrm {d}x\,\mathrm {d}y}\\&\quad \precsim C(D_2)\left( \Vert u-(u)_{D_2}\Vert _{L^{\frac{2}{1-2s}}(D_2)}[\varphi ]_{W^{\varepsilon ,\frac{2}{2s+1}}(D_2)} + \Vert u\Vert _{L^1(D_2)} [\varphi ]_{W^{\varepsilon ,\frac{2}{2s+1}}(D_2)} \right) . \end{aligned} \end{aligned}$$

We also have

B.5 $$\begin{aligned} \int _{D_2} |\varphi (y)|\int _{D_2} \frac{|u(x)-u(y)|}{|x-y|}{\,\mathrm {d}x\,\mathrm {d}y} \precsim \Vert \varphi \Vert _{L^2(D_2)}\, [u]_{W^{s,2}(D_2)}. \end{aligned}$$

Combining (B.3) with (B.4) (in which we use Poincarè inequality) and (B.5), we obtain

B.6 $$\begin{aligned} \begin{aligned}&\int _{D_2} \int _{D_2} \frac{(\eta (x)-\eta (y)) \left( u(y) \varphi (x) - u(x) \varphi (y) \right) }{|x-y|^{2}}{\,\mathrm {d}x\,\mathrm {d}y}\\&\quad \precsim \Vert \eta \Vert _{\mathrm{Lip\,}}\left( \Vert u\Vert _{L^1(D_2)} + [u]_{W^{s,2}(D_2)} \right) \, \left( \Vert \varphi \Vert _{L^2(D_2)} + [\varphi ]_{W^{\varepsilon ,\frac{2}{2s+1}}(D_2)} \right) . \end{aligned} \end{aligned}$$

For the second term of (B.2), observe that for $x \in D_1$ and $y \in D \setminus D_2$ we have $|x-y| \approx 1 + |y|$, so we have

B.7 $$\begin{aligned} \begin{aligned}&2\left| \int _{D_1} \int _{D \setminus D_2} \frac{(\eta (x)-\eta (y)) \left( u(y) \varphi (x) - u(x) \varphi (y) \right) }{|x-y|^{2}}{\,\mathrm {d}y\,\mathrm {d}x} \right| \\&\quad \precsim \Vert \eta \Vert _{L^\infty }\left| \int _{D_1} \int _{D \setminus D_2} \frac{\left| u(y) \right| \left| \varphi (x) \right| + \left| u(x) \right| \left| \varphi (y) \right| }{1+|y|^{2}}{\,\mathrm {d}y\,\mathrm {d}x} \right| \\&\quad \precsim \Vert \eta \Vert _{L^\infty }\Vert u\Vert _{L^1+L^\infty (D)}\, \Vert \varphi \Vert _{L^1+L^\infty (D)}. \end{aligned} \end{aligned}$$

Thus, by (B.2), (B.6), and (B.7) we get

B.8 $$\begin{aligned} |\mathcal {G}_1(u,\varphi )| \precsim \left( \Vert u\Vert _{L^1+L^\infty (D)}+[u]_{W^{s,2}(D_2)} \right) \left( \Vert \varphi \Vert _{L^2+L^\infty (D)} + [\varphi ]_{W^{\varepsilon ,\frac{2}{2s+1}}(D_2)} \right) . \end{aligned}$$

${{\textit{Estimate of }\mathcal {G}_2:}}$ Similarly as in (B.7), if $x \in D_1$ and $y \in {\mathbb {R}}\setminus D$ we have $|x-y| \approx 1+|y|$, and thus

$$\begin{aligned}&|\mathcal {G}_2(u,\varphi )| \precsim \Vert \eta u\Vert _{L^2(D)}\left( \Vert \varphi \Vert _{L^2({D_1})} + \Vert \varphi \Vert _{L^1{+L^\infty }({\mathbb {R}})} \right) \\&\quad \precsim \Vert u\Vert _{L^2(D_1)}\left( \Vert \varphi \Vert _{{L^2+L^\infty }(D)} + \Vert \varphi \Vert _{L^1 + L^\infty ({\mathbb {R}})} \right) . \end{aligned}$$

${{\textit{Estimate of }\mathcal {G}_3:}}$ Using the support of *v*, observing again that $|x-y| \succsim 1+|y|$ if $y \in {\mathbb {R}}\setminus D_2$ and $x \in D_1$, we get

$$\begin{aligned} \begin{aligned}&|\mathcal {G}_3(u,\varphi )|\\&\quad \precsim \Vert \Omega \Vert _{L^2(\bigwedge \nolimits ^1_{od} D)} \left( \int _{D \setminus D_2} \int _{D_1} |u(x)|^2\, |\varphi (x)|^2\, \frac{\,\mathrm {d}x\,\mathrm {d}y}{1+|y|^{2}} \right. \\&\qquad \left. + \int _{D_1} \int _{D \setminus D_2} |u(y)|^2 |\varphi (x)|^2\, \frac{\,\mathrm {d}x\,\mathrm {d}y}{1+|x|^{2}}\right) ^{\frac{1}{2}}\\&\quad \precsim \Vert \Omega \Vert _{L^2(\bigwedge \nolimits ^1_{od} D)}\, \left( \Vert u\varphi \Vert _{L^2(D_1)} + \Vert \varphi \Vert _{L^2{+L^\infty }(D)}\, \Vert u\Vert _{L^2(D_1)} \right) \\&\quad \precsim \Vert \Omega \Vert _{L^2(\bigwedge \nolimits ^1_{od} D)}\, \left( \Vert u\Vert _{L^1(D_1)} \Vert \varphi \Vert _{L^2(D_1)} + \Vert u-(u)_{D_1}\Vert _{L^{\frac{2}{1-2s}}(D_1)}\, \Vert \varphi \Vert _{L^{\frac{1}{s}}(D_1)}\right. \\&\qquad \left. + \Vert \varphi \Vert _{L^2{+L^\infty }(D)}\, \Vert u\Vert _{L^2(D_1)}\right) \\&\quad \precsim \Vert \Omega \Vert _{L^2(\bigwedge \nolimits ^1_{od} D)}\, \left( \Vert u\Vert _{L^1(D_1)} \Vert \varphi \Vert _{L^2(D_1)} + [u]_{W^{s,2}(D_1)}\, \Vert \varphi \Vert _{L^{\frac{1}{s}}(D_1)}\right. \\&\qquad \left. + \Vert \varphi \Vert _{L^2{+L^\infty }(D)}\, \Vert u\Vert _{L^2(D_1)}\right) \\&\quad \precsim \Vert \Omega \Vert _{L^2(\bigwedge \nolimits ^1_{od} D)}\, \left( \Vert u\Vert _{L^2(D_1)} + [u]_{W^{s,2}(D_1)} \right) \left( \Vert \varphi \Vert _{L^{\frac{1}{s}}(D_1)} + \Vert \varphi \Vert _{L^2{+L^\infty }(D)} \right) . \end{aligned} \end{aligned}$$

This argument works for any $s \in (0,\frac{1}{2})$.

$${{\textit{Estimate of }\mathcal {G}_4:}}$$ We have

$$\begin{aligned} |\mathcal {G}_4(u,\varphi )| \precsim \Vert \Omega \Vert _{L^2(\bigwedge \nolimits ^1_{od} D)} \left( \int _{D} \int _{D} |u(y) d_{\frac{1}{2}}\eta (x,y)\varphi (x)|^2\, \frac{\,\mathrm {d}x\,\mathrm {d}y}{|x-y|} \right) ^{\frac{1}{2}}. \end{aligned}$$

Now observe that $|d_{\frac{1}{2}} \eta (x,y)|^2 \le \Vert \eta \Vert _{\mathrm{Lip\,}}^2 |x-y|$, thus

$$\begin{aligned} \left( \int _{D} \int _{D} |u(y) d_{\frac{1}{2}}\eta (x,y)\varphi (x)|^2\, \frac{\,\mathrm {d}x\,\mathrm {d}y}{|x-y|} \right) ^{\frac{1}{2}} \precsim \Vert u\Vert _{L^2(D)}\, \Vert \varphi \Vert _{L^2(D)}. \end{aligned}$$

On the other hand

$$\begin{aligned} \left( \int _{D} \int _{D} |u(y) d_{\frac{1}{2}}\eta (x,y)\varphi (x)|^2\, \frac{\,\mathrm {d}x\,\mathrm {d}y}{|x-y|} \right) ^{\frac{1}{2}} \precsim [\eta ]_{W^{\frac{1}{2},2}}\Vert u\Vert _{L^\infty (D)}\, \Vert \varphi \Vert _{L^\infty (D)}. \end{aligned}$$

We also have

$$\begin{aligned}&\left( \int _{D} \int _{D} |u(y) d_{\frac{1}{2}}\eta (x,y)\varphi (x)|^2\, \frac{\,\mathrm {d}x\,\mathrm {d}y}{|x-y|} \right) ^{\frac{1}{2}} \\&\quad \precsim \Vert u\Vert _{L^\infty (D)}\, \Vert \varphi \Vert _{L^2(D)}\, \sup _{x\in D} \left( \int _{D} \frac{|\eta (x)-\eta (y)|^2}{|x-y|^2}\,\mathrm {d}y \right) ^{\frac{1}{2}} \end{aligned}$$

and

$$\begin{aligned} \sup _{x\in D} \left( \int _{D} \frac{|\eta (x)-\eta (y)|^2}{|x-y|^2}\,\mathrm {d}y \right) ^{\frac{1}{2}} \precsim \Vert \eta \Vert _{\mathrm{Lip\,}}. \end{aligned}$$

Thus, combining the estimates on $\mathcal {G}_4$ we obtain

$$\begin{aligned} |\mathcal {G}_4(u,\varphi )| \precsim \Vert \Omega \Vert _{L^2(\bigwedge \nolimits ^1_{od} D)} \Vert u\Vert _{L^2+L^\infty (D)} \Vert \varphi \Vert _{L^2+L^\infty (D)}. \end{aligned}$$

$$\square $$

## Appendix C: A Sobolev inequality

### Theorem C.1

Let $s \in (0,1)$, $p,q \in (1,\infty )$ and $f \in L^p({\mathbb {R}}^n)$ then

1. $$\begin{aligned}{}[f]_{{\dot{F}}^s_{p,q}({\mathbb {R}}^n)} \precsim [f]_{W^{s}_{p,q}({\mathbb {R}}^n)}; \end{aligned}$$
2. if $$p > \frac{nq}{n+sq}$$ then
  $$\begin{aligned}{}[f]_{W^{s}_{p,q}({\mathbb {R}}^n)} \precsim [f]_{{\dot{F}}^s_{p,q}({\mathbb {R}}^n)}. \end{aligned}$$

The constants depend on *s*, *p*, *q*, *n* and are otherwise uniform.

While characterizations such as Theorem C.1 are well known for Besov spaces, for Triebel spaces this seems to have been known only for $q=p$ (where it follows from the Besov-space characterization), $q=2$ where it is a result due to Stein and Fefferman, [12, 33]. It was also known “for large s” [34, Section 2.5.10]. Although a conjecture that Theorem C.1 holds is very natural, quite surprisingly, to the best of our knowledge, the first time Theorem C.1 has been proven was recently by Prats and Saksman [26, Theorem 1.2] (see also [25] for further development), but see also [32, 35].

### Corollary C.2

Let $s \in (0,1)$, $t \in (s,1)$ and $p,p^*\in (1,\infty )$ where

C.1 $$\begin{aligned} s-\frac{n}{p^*} = t-\frac{n}{p}. \end{aligned}$$

If $q \in (1,\infty )$ such that $p^*> \frac{nq}{n+sq}$ we have

$$\begin{aligned} \Vert |\mathcal {D}_{s,q} f|\Vert _{L^{p^*}({\mathbb {R}}^n)} \precsim \Vert (-\Delta )^{\frac{t}{2}} f\Vert _{L^p({\mathbb {R}}^n)}. \end{aligned}$$

More precisely, in terms of Lorentz spaces we have for any $r \in [1,\infty ]$,

$$\begin{aligned} \Vert |\mathcal {D}_{s,q} f|\Vert _{L^{(p^*,r)}({\mathbb {R}}^n)} \precsim \Vert (-\Delta )^{\frac{t}{2}} f\Vert _{L^{(p,r)}({\mathbb {R}}^n)}. \end{aligned}$$

### Proof

From Theorem C.1 we have

$$\begin{aligned} \Vert |\mathcal {D}_{s,q} f|\Vert _{L^{p^*}({\mathbb {R}}^n)} \approx [f]_{F^s_{p^*,q}({\mathbb {R}}^n)}. \end{aligned}$$

We recall the Sobolev-embedding theorem for Triebel–Lizorkin spaces ${\dot{F}}^t_{p,\tilde{q}} \hookrightarrow {\dot{F}}^{{s}}_{{p^*},q}$ for any $q,\tilde{q} \in (1,\infty )$ and $s,\, t,\, p,\,p^*$ satisfying (C.1) (see, e.g., [34, Theorem 2.7.1 (ii)]). Thus,

$$\begin{aligned} \Vert |\mathcal {D}_{s,q} f|\Vert _{L^{p^*}({\mathbb {R}}^n)} \precsim [f]_{F^t_{p,2}({\mathbb {R}}^n)} \precsim \Vert (-\Delta )^{\frac{t}{2}} f\Vert _{L^{p}({\mathbb {R}}^n)}. \end{aligned}$$

As for the Lorentz-space estimate, we can argue by real interpolation. Indeed, fix $s,q,p,p^*$. Observe that $f \mapsto |\mathcal {D}_{s,q} f|$ is a sublinear operator. We can find $p_1< p < p_2$ such that $p_1$ and $p_2$ are still admissible, and thus we have

$$\begin{aligned} \Vert |\mathcal {D}_{s,q} f|\Vert _{L^{p_i^*}({\mathbb {R}}^n)} \precsim [f]_{F^t_{p,2}({\mathbb {R}}^n)} \precsim \Vert (-\Delta )^{\frac{t}{2}} f\Vert _{L^{p_i}({\mathbb {R}}^n)} \quad i=1,2. \end{aligned}$$

From real interpolation we now obtain the Lorentz space claim. $\square$

## Appendix D: A sequence of cut-off functions in the critical Sobolev space

For readers convenience we present here a proof of a well-known result, which essentially says that in the critical Sobolev space a point has zero capacity. See for example [1, Theorem 5.1.9]; compare also with a similar construction [23, Lemma 3.2].

### Lemma D.1

There exists a sequence of functions with the following properties:

$\{\zeta _\ell \}_{\ell \in {{\mathbb {N}}}} \subset C_c^\infty ({\mathbb {R}},[0,1])$ and for all $\ell \in {{\mathbb {N}}}$ we have

D.1 $$\begin{aligned} \zeta _\ell \equiv 1 \text { on } B_{\rho _\ell }(x_0), \quad \zeta _\ell \equiv 0 \text { outside } B_{R_\ell }(x_0), \quad \text { and }\lim _{\ell \rightarrow \infty }[\zeta _\ell ]_{W^{\frac{1}{2},2}({\mathbb {R}})}=0 \end{aligned}$$

for a sequence of radii $0<\rho _\ell <R_\ell \rightarrow 0$ as $\ell \rightarrow \infty$.

### Proof

Let $f(x) = \log \log \left( 1 + \frac{1}{|x|^2} \right) \in W^{1,2}(B^2_1,{\mathbb {R}})$ be an unbounded function. We define

$$\begin{aligned} \tilde{Z}_k(x) :=\left\{ \begin{array}{ll} 1 &{} \text { if } f(x)\ge k+1,\\ f(x) - k &{} \text { if } k\le f(x)\le k+1,\\ 0 &{} \text { if } f(x)< k. \end{array} \right. \end{aligned}$$

Then,

$$\begin{aligned} \nabla \tilde{Z}_k(x) :=\left\{ \begin{array}{ll} 0 &{} \text { if } f(x)\ge k+1,\\ \nabla f(x) &{} \text { if } k\le f(x)\le k+1,\\ 0 &{} \text { if } f(x)< k. \end{array} \right. \end{aligned}$$

The support of $\nabla \tilde{Z}_k$ is the set

$$\begin{aligned} B_k:=\left\{ x\in B_1^2:A_{k+1}\le |x| \le A_k\right\} , \end{aligned}$$

where

$$\begin{aligned} A_k = \sqrt{\frac{1}{e^{e^{k}}-1}}, \quad A_{k+1}\le A_k,\quad \text { and } \quad \lim _{k\rightarrow \infty } A_k =0. \end{aligned}$$

Now,

$$\begin{aligned} \int _{B_1}|\nabla \tilde{Z}_k|^2 \,\mathrm {d}x= \int _{A_{k+1}\le |x|\le A_k} |\nabla \tilde{Z}_k|^2 \,\mathrm {d}x \xrightarrow {k\rightarrow \infty }0, \end{aligned}$$

which follows from the fact that $\nabla \tilde{Z}_k\in L^2(B_1^2)$ and that $|\{x\in B_1^2:A_{k+1}\le |x| \le A_k\}|$ shrinks to zero.

Thus, we obtained a sequence of functions for which

$$\begin{aligned} \tilde{Z}_k\equiv 1 \text { on } B_{A_{k+1}},\quad \tilde{Z}_k\equiv 0 \text { outside } B_{A_k}, \quad \text { and } \lim _{k\rightarrow \infty }\Vert \nabla \tilde{Z}_k\Vert _{L^2(B^2_1)}=0. \end{aligned}$$

By extending by zero we obtain a sequence $Z_k\in W^{1,2}({\mathbb {R}}^2_+)$ with the properties

D.2 $$\begin{aligned} {Z}_k\equiv 1 \text { on } B_{A_{k+1}},\quad {Z}_k\equiv 0 \text { outside } B_{A_k}, \quad \text { and } \lim _{k\rightarrow \infty }\Vert \nabla {Z}_k\Vert _{L^2({\mathbb {R}}^2_+)}=0. \end{aligned}$$

Defining now $\zeta _k:=Z_k\big |_{{\mathbb {R}}}$ in the trace sense, we obtain by the trace inequality, [13]

$$\begin{aligned}{}[\zeta _k]_{W^{\frac{1}{2},2}({\mathbb {R}})} \precsim \Vert \nabla Z_k\Vert _{L^2({\mathbb {R}}^2_+)} \xrightarrow {k\rightarrow \infty }0. \end{aligned}$$

Approximating $\{\zeta _k\}_{k\in {{\mathbb {N}}}}$ by smooth functions, we obtain the desired sequence. $\square$
